# Modulation of the functional interfaces between retroviral intasomes and the human nucleosome

**DOI:** 10.1128/mbio.01083-23

**Published:** 2023-06-29

**Authors:** E. Mauro, D. Lapaillerie, C. Tumiotto, C. Charlier, F. Martins, S. F. Sousa, M. Métifiot, P. Weigel, K. Yamatsugu, M. Kanai, H. Munier-Lehmann, C. Richetta, M. Maisch, J. Dutrieux, J. Batisse, M. Ruff, O. Delelis, P. Lesbats, V. Parissi

**Affiliations:** 1 Fundamental Microbiology and Pathogenicity Lab (MFP), UMR 5234 CNRS-University of Bordeaux, SFR TransBioMed, Bordeaux, France; 2 Viral DNA Integration and Chromatin Dynamics Network (DyNAVir), Bordeaux, France; 3 Nantes Université, CNRS, US2B, UMR 6286 and CHU Nantes, Inserm, CNRS, SFR Bonamy, IMPACT Platform, Nantes, France; 4 UCIBIO@REQUIMTE, BioSIM Departamento de Biomedicina, Faculdade de Medicina da Universidade do Porto, Alameda Professor Hernâni Monteiro, Porto, Portugal; 5 Graduate School of Pharmaceutical Sciences, The University of Tokyo, Tokyo, Japan; 6 Institut Pasteur, Unité de Chimie et Biocatalyse, CNRS UMR 3523, Paris, France; 7 LBPA, ENS Paris-Saclay, CNRS UMR8113, IDA FR3242, Université Paris-Saclay, Cachan, France; 8 Université Paris Cité, Institut Cochin, INSERM U1016, CNRS, UMR8104, Paris, France; 9 Département de Biologie Structurale intégrative, IGBMC (Institut de Génétique et de Biologie Moléculaire et Cellulaire), UDS, U596 INSERM, UMR7104, CNRS, Strasbourg, France; Columbia University Medical Center, New York, New York, USA

**Keywords:** retrovirus, host chromatine, HIV-1, integration, nucleosome

## Abstract

Infection by retroviruses as HIV-1 requires the stable integration of their genome into the host cells. This process needs the formation of integrase (IN)-viral DNA complexes, called intasomes, and their interaction with the target DNA wrapped around nucleosomes within cell chromatin. To provide new tools to analyze this association and select drugs, we applied the AlphaLISA technology to the complex formed between the prototype foamy virus (PFV) intasome and nucleosome reconstituted on 601 Widom sequence. This system allowed us to monitor the association between both partners and select small molecules that could modulate the intasome/nucleosome association. Using this approach, drugs acting either on the DNA topology within the nucleosome or on the IN/histone tail interactions have been selected. Within these compounds, doxorubicin and histone binders calixarenes were characterized using biochemical, *in silico* molecular simulations and cellular approaches. These drugs were shown to inhibit both PFV and HIV-1 integration *in vitro*. Treatment of HIV-1-infected PBMCs with the selected molecules induces a decrease in viral infectivity and blocks the integration process. Thus, in addition to providing new information about intasome-nucleosome interaction determinants, our work also paves the way for further unedited antiviral strategies that target the final step of intasome/chromatin anchoring.

**IMPORTANCE:**

In this work, we report the first monitoring of retroviral intasome/nucleosome interaction by AlphaLISA. This is the first description of the AlphaLISA application for large nucleoprotein complexes (>200 kDa) proving that this technology is suitable for molecular characterization and bimolecular inhibitor screening assays using such large complexes. Using this system, we have identified new drugs disrupting or preventing the intasome/nucleosome complex and inhibiting HIV-1 integration both *in vitro* and in infected cells. This first monitoring of the retroviral/intasome complex should allow the development of multiple applications including the analyses of the influence of cellular partners, the study of additional retroviral intasomes, and the determination of specific interfaces. Our work also provides the technical bases for the screening of larger libraries of drugs targeting specifically these functional nucleoprotein complexes, or additional nucleosome-partner complexes, as well as for their characterization.

## INTRODUCTION

Retroviruses must integrate their viral DNA (vDNA) into the host cell genome to achieve productive infection. In infected cells, the preintegration complex (PIC) is responsible for this process. The PIC is composed of the viral intasome and cellular cofactors including, in the case of lentiviruses, LEDGF/p75, barrier-to-autointegration factor, high-mobility group protein A1, and histones [for a recent review, see reference (1)]. *In vitro,* the intasome nucleoprotein complex, which is composed of an oligomer of integrase (IN) engaging the ends of the vDNA, is sufficient for catalyzing integration. The first intasome that has been structurally characterized is the spumaretroviral prototype foamy virus (PFV) intasome, using X-ray crystallography, revealing a complex composed of four IN protomers (2). It has been generally believed that this tetramer is also sufficient to catalyze the integration of other retroviruses. However, elucidation of intasome structures from other retroviral genera revealed a different organization of these complexes: intasomes from the alpharetrovirus RSV and the betaretrovirus MMTV are composed of an oligomer of 8 INs (3), while the lentivirus MVV and HIV-1 may contain 12–16 INs (4, 5). More recently, another lentiviral intasome, SIV, was revealed to be composed of 12 INs (6). Despite the apparent heterogeneity of these complexes, they share a common core structure called the conserved intasome core, which exhibits a similar organization to the tetrameric PFV intasome. However, these differences in the global quaternary structure also suggest possible differences in their interfaces with cellular partners and nucleosomal substrates that may account for previously observed differences in their sensitivity toward chromatin structure (7 - 9) and, thus, at least partially, in their preference for host insertion sites. This is further supported by a recent report showing distinct *in vitro* interactions between chromosomes and either the PFV intasome or the HIV-1 intasome (9). These observations may be in agreement with the distinct cellular insertion sites found for the different retroviruses. Indeed, retroviruses do not integrate randomly into the host cell genome. Numerous cellular cofactors and viral proteins have been shown to be involved in tethering the intasome to specific regions of chromatin, depending on the retrovirus. However, the final target of the integration remains the nucleosome, and thus, its complex with incoming intasome constitutes the relevant antiviral target in infected cells.

Nucleosomes are composed of cellular DNA (147 bp) wrapped around an octamer of histone proteins (H2A, H2B, H3, and H4, with two copies of each of the histones). To date, structural details of the intasome-nucleosome complex are available only for PFV (10, 11). These structures highlighted a specific positioning of the intasome on the nucleosome, involving several interfaces with three IN subunits, both gyres of the nucleosomal DNA, the core of H2B, and the tail of H2A. Similar requirements for direct contact with histone tails have been reported for lentiviral models such as HIV-1 (12, 13). Even if the intasome-nucleosome complex displays multiple interfaces, point mutations in the IN targeting each of these interfaces could drastically reduce both the interaction with the nucleosome and the chromosomes and the cellular integration efficiency (9, 10, 12), pointing to them as possible candidates for therapeutic agents or molecular chemical tools.

Thus, the multiple intasome/nucleosome interfaces determine the formation of the active complexes. A deeper analysis of these interfaces is needed for a better understanding of the dynamic assembly of these supramolecular complexes. With this purpose, we used the AlphaLISA approach to monitor the intasome/nucleosome complex. A pharmacology approach was then used to select small molecules targeting the specific PFV intasome/nucleosome complex that was able to scan the functional interfaces and modulate their assembly. Several drugs have been identified based on their capability to dissociate the complex by acting either on IN/histone or histone/DNA interactions or on DNA topology. Their mechanism of action was further characterized, leading us to identify new molecular modulators of the intasome/nucleosome functional interfaces. These new drugs were found active on HIV-1 integration both *in vitro* and infected cells validating them as potential new lead compounds for the development of therapeutic agents affecting intasome/nucleosome complex stability.

## RESULTS

### Monitoring the functional PFV intasome/nucleosome complexes using AlphaLISA technology

**Fig 1:**
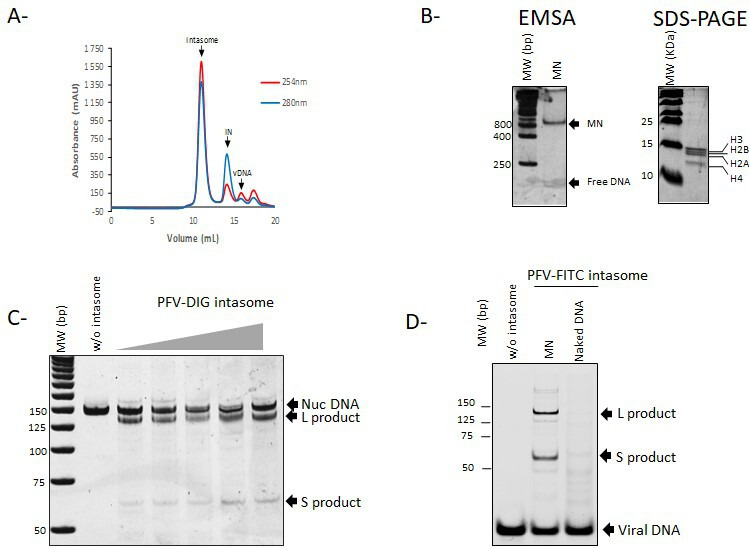
Assembly of functional PFV intasome and human nucleosome. The PFV intasome was assembled as described in Materials and Methods and subsequently purified by size exclusion chromatography (**A**). Protein and DNA elution was monitored by 280 and 254 nm absorbance, respectively. MN was assembled by salt dialysis protocol using 5′ biotinylated 601 Widom sequence and human octamers. The structure of the nucleosome was checked by native 8% polyacrylamide gel and 15% SDS-PAGE (**B**). The functionality of the intasome complex was checked by *in vitro* concerted integration assay performed on the assembled MN using 0–710 nM of intasome and 100 ng of MN for 30 nM. Integration was monitored by 1% agarose gel stained with SybrSafe (**C**). Single-site integration results in the formation of long (**L**) and short (**S**) products. Integration catalyzed by FITC-coupled intasome is monitored directly onto gel (**D**).

To set up an AlphaLISA approach to monitor the complex formed between the retroviral intasome and the human mononucleosome (MN), we first used the well-defined PFV intasome. The PFV intasome was assembled following the reported procedure (10) using its cognate vDNA fused to a DIG tag at the 5′ end of the viral ODN mimicking the final ends of the PFV LTR U5 sequence. As shown in Fig. 1A, the elution profile of the intasome was consistent with published works. The MN was assembled using purified human octamers and a biotinylated 601 Widom sequence (14) following typical salt dialysis procedures, as previously used in the laboratory (9) and described in Materials and Methods. Each MN assembly was checked using native 8% polyacrylamide gel, and the lack of contamination with hexa- and tetrasomes was checked by the detection of equal amounts of histones in SDS-PAGE (Fig. 1B). Stable native biotinylated MNs obtained with only poorly detectable amounts of free DNA were used for functional and interaction assays. The functionality of the intasome/nucleosome complexes was controlled by a typical concerted integration assay and is shown in Fig. 1C. A major single integration product resulting from a unique docking position of the intasome onto the MN could be detected, leading to the formation of large (L) and small (S) bands resulting from the previously observed PFV insertion site (10). To better monitor the integration products and fully validate the functionality of the purified intasome, we also assembled an intasome with FITC-coupled vDNA. No change in the assembly profile was observed when PFV FITC-IN was used (data not shown). As reported in Fig. 1D, the FITC intasome was also found to be active on MNs, generating the expected integration products and confirming the functional assembly of the complex. An integration assay performed on naked 601 DNA also showed that MN was a preferential substrate and that integration sites detected on MN were specific to this structure. All these data fully confirmed that functional intasomes and MN nucleocomplexes could be assembled using differently tagged partners, allowing their use in AlphaLISA and integration assays.

**Fig 2:**
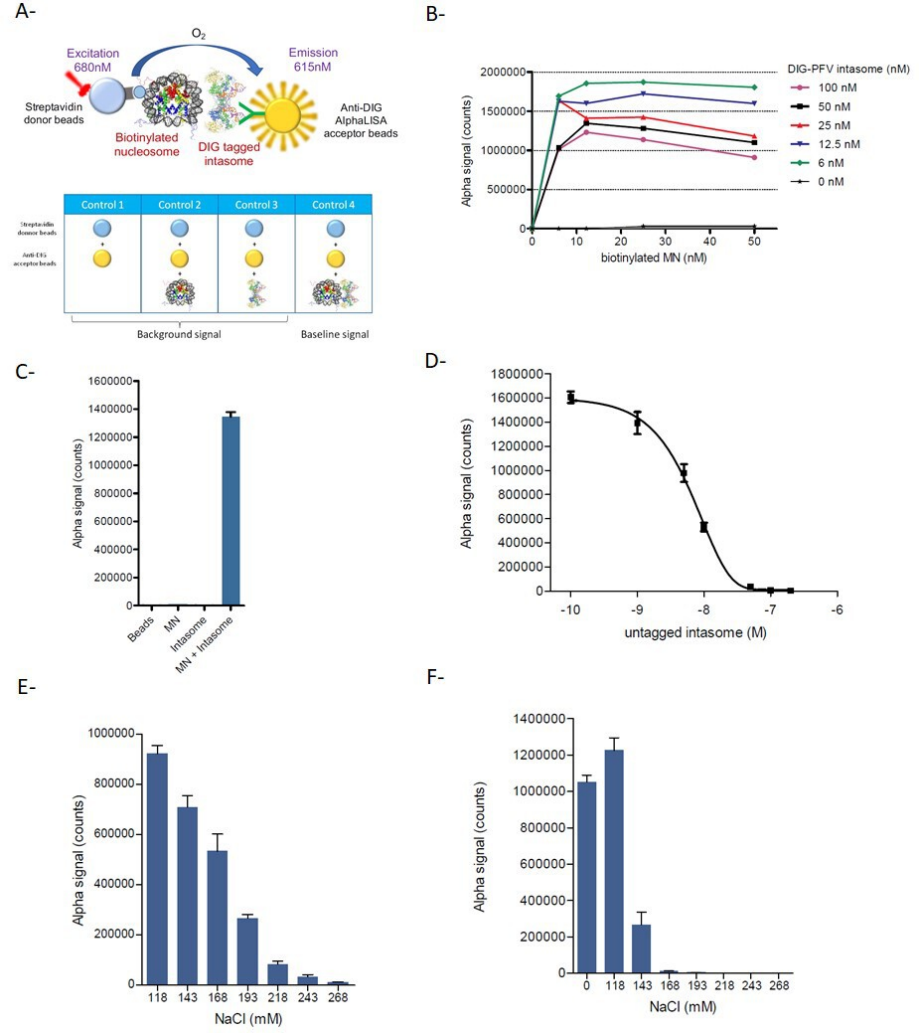
Setup of the intasome/nucleosome AlphaLISA assay. The principle of the AlphaLISA approaches using human biotinylated MN and DIG-tagged PFV intasome as well as the control conditions are schematized in (**A**). The AlphaLISA interaction signal (AU) was monitored using increasing concentrations of each partner, and data are reported in (**B**) as a representative experiment. Comparison of the interaction signal obtained with both partners (1 nM), each partner, or beads alone is reported in (**C**) as the mean of three independent experiments ± SD. Competition experiment performed with increasing concentration of untagged intasome is reported in (**D**) as the mean of three independent experiments ± SD. The Alphascreen interaction signal (AU) was also monitored using the increasing concentration of NaCl using 3 nM of intasome and 3 nM of either nucleosome (**E**) or naked DNA (**F**), and data are reported as the mean of three independent experiments ± SD.

AlphaLISA technology was employed as shown in Fig. 2A using anti-DIG acceptor and streptavidin donor beads. The cross-titration experiments shown in Fig. 2B indicate that a strong intasome/nucleosome interaction signal could be detected (>10^6^ counts), even at low concentrations of each partner (6–100 nM). Additionally, titration experiments reported in SI1 showed that a robust AS signal >10^6^ counts could still be obtained with 1 nM of each intasome and MN partner. These optimal conditions were, thus, used in the following parts of the work. Control experiments, shown in Fig. 2A, performed with similar amounts of beads, MN, or intasome alone confirmed the specificity of the interaction signal observed with both partners (Fig. 2C). As reported in Fig. 2D, the addition of free unlabeled intasome led to an inhibition of the DIG-intasome/Biotin-MN interaction, showing a typical sigmoid competition curve. The fitting of the curve allowed us to calculate the apparent IC_50_ of 27 nM for the complex formed under these conditions. These competitive displacement measurements confirm the specificity of this AlphaLISA binding assay and validate its use to identify compounds that modulate intasome/MN interactions. Since the intasome/MN interactions involved multiple interfaces, including protein/protein and protein/DNA interactions, we compared the AlphaLISA signal obtained with the intasome and either the MN or the corresponding 147 bp 601 DNA. A similar interaction signal was detected in the presence of MN and DNA (Fig. 2E and F) when using 100 mM NaCl concentrations. However, the sensitivity of the interactions to increasing salt concentrations was found to be different. Indeed, the interaction signal between the intasome and DNA decreased more rapidly than that between the intasome and MN. While a significant interaction signal was still detected at 200 mM NaCl in the presence of MN, the AS signal completely dropped to the zero baseline in the presence of DNA. This confirmed that at concentrations below 200 mM, protein/protein interactions participate in the nucleocomplex interactions in addition to protein/DNA interactions, while at salt concentration upper than 200 mM, the protein/protein interactions are the main components of the AS interaction signal.

Taken together, these data indicate that the functional intasome/MN complex can be monitored using the AlphaLISA approach, leading to a robust interaction signal even when using low amounts of each partner. This allowed us to further use this system for validating the feasibility of drug screening assays and select compounds that may modulate the intasome/nucleosome interaction.

### Selection of drugs affecting the PFV intasome/nucleosome association

**Fig 3:**
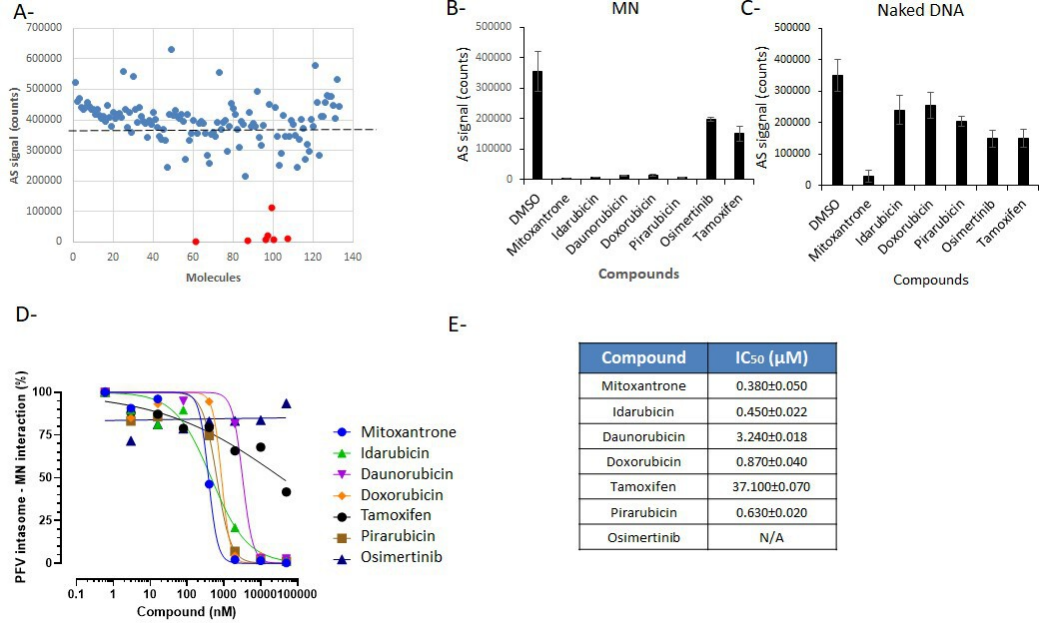
Selection of drugs preventing the intasome/nucleosome association from the ONCOSET NIH library. The 133 compounds of the OncoSET library have been tested at 25 µM on the PFV intasome/nucleosome AlphaLISA interaction (**A**). The selected compounds indicated as red bullets have been re-tested at 10 µM using the MN (**B**) or the naked 601 DNA (**C**). The validated drugs have been further tested using increasing concentrations in intasome/nucleosome AS assay (**D**). The calculated IC_50_ values are reported in (**E**). Data are reported as the mean of two to three independent experiments ± SD.

Since most of the drug assays require the use of dimethyl sulfoxide (DMSO), we first tested the robustness of our system toward concentrations of this solvent. As shown in SI1**,** a decrease in the AS signal was observed only above 2.5% DMSO, and the signal was decreased by 2 at 10% and 6 at 20%. We concluded that the limit concentration of DMSO usable in this assay was 2.5%. Then, the NIH OncoSET library [see description in Materials and Methods (15, 16)] was screened. This library has been chosen since it contains up to 133 FDA-approved compounds targeting chromatin-associated proteins and DNA topology that constitute determinants of the intasome/nucleosome complex. Screening was initially performed using 25 µM of each drug in 384-well plates and previously optimized interaction conditions with 1 nM of each partner as described in Materials and Methods. Negative controls (beads, MN, and intasome alone) were systematically added to the screen. As reported in Fig. 3A, among the 133 drugs, seven compounds were selected as inducing a decrease of at least 50% in the AS signal: mitoxanthrone, idarubicin, daunorubicin, doxorubicin, pirarubicin, tamoxifen, and osimertinib.

Each selected drug was retested at 10 µM for its effect on the intasome/nucleosome interaction on the AS signal, and as reported in Fig. 3B, the results confirmed the strong inhibitory effect observed for mitoxanthrone, idarubicin, daunorubicin, doxorubicin, and pirarubicin, while tamoxifen showed a 50–60% decrease in the AS signal, and osimertinib showed a 30–40% decrease in the AS signal. To better ascertain whether the selected drugs specifically target the intasome/nucleosome complex, we also tested them on the complex form between the naked 601 DNA and the intasome. As shown in Fig. 3C, most of the drugs were found less efficient in blocking the intasome/naked DNA interaction except the mitoxantrone, osimertinib, and tamoxifen. As reported in Fig. 3D, dose-response sigmoid curves were observed for all drugs in intasome/nucleosome AS assay except for tamoxifen, which showed only a slight inhibition effect, and osimertinib, which showed no inhibition in the concentration range used and was, thus, excluded from further analysis. The IC_50_ values of the selected drugs reported in Fig. 3E were between 0.383 and 37.059 µM. In order to determine whether the selected compounds may interfere with the fluorescent AlphaLISA assay due to autofluorescence or quenching properties, we tested the drugs on the typical counter assay described in SI2. Among the selected drugs, mitoxanthrone induced a strong decrease in the AS signal in this control experiment suggesting that it may constitute a false positive in the initial screen. To confirm this and identify possible inhibitors of the retroviral integration, all the selected molecules were tested in *in vitro* integration assays.

### Selection of compounds specifically inhibiting *in vitro* PFV nucleosomal integration

**Fig 4:**
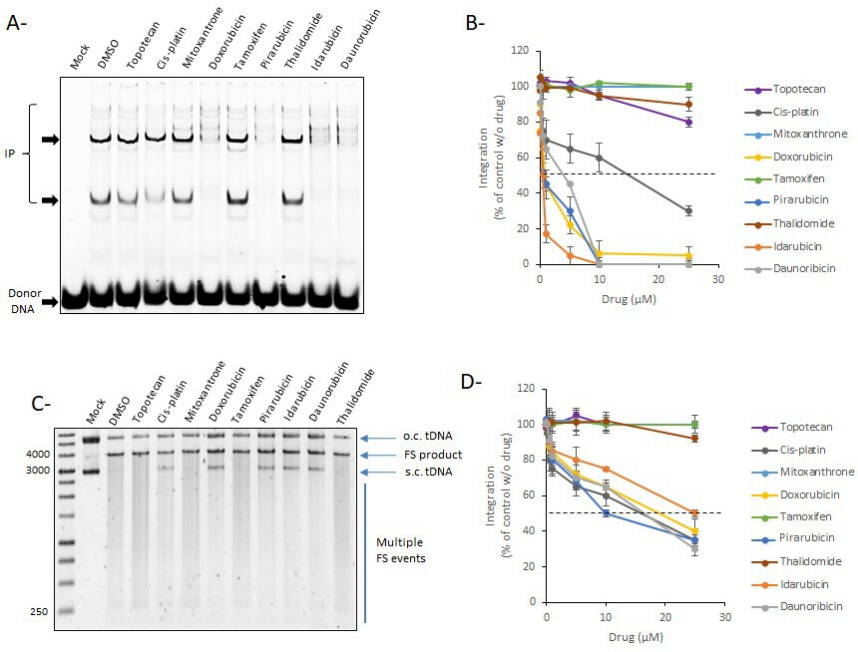
Effect of the selected compounds on *in vitro* PFV integration. The compounds selected in Fig. 3 have been tested in typical concerted integration onto mononucleosome using 30 nM of FITC PFV intasome and 200 ng of MN (100 ng of histones assembled on 100 ng of DNA) (**A and B**), naked DNA plasmid (**C**), naked 147 bp Widom 601 fragment (**D**), and increasing concentrations of drugs (0–25 µM). (**A**) and (**C**) show representative experiments performed at 10 µM of drugs, and (**B**) and (**D**) show the means from three independent experiments ± SD. The dotted line indicates the 50% of integration. o.c., open circular; s.c., supercoiled; FS, full-site integration product; IP, integration products.

To determine whether the inhibition of the intasome/nucleosome interaction in the AlphaLISA assay could be associated with an inhibition of the functional integration reaction, the selected drugs were tested in a typical *in vitro* concerted integration assay. As shown in Fig. 4, idarubicin, daunorubicin, doxorubicin, and pidorubicin, which are the most efficient drugs for inhibiting the AS interaction signal, were also able to inhibit PFV integration into MNs. Tamoxifen was inefficient in inhibiting integration, which is in agreement with its poor effect on the intasome/nucleosome interaction detected in AS. Mitoxanthrone did not show any inhibitory effect on integration despite its negative effect on the AS signal confirming that it may be a false-positive, as shown by counter assay (see SI2). Control experiments performed with additional drugs from the library but not selected by AS, such as the DNA intercalating drug thalidomide and the topoisomerase I inhibitor topotecan, did not show any effect, confirming the specific activity of the selected drugs. The intercalating cisplatin agent was also used as a positive control of inhibition due to its strong DNA intercalating potency and showed only a slight inhibition of integration into nucleosome. Quantitative and comparative integration assays performed using immobilized nucleosomes allowed us to better determine the inhibitory potency of the drugs (Fig. 4B). In this assay, the selected drugs showed IC_50_ values of MN integration between 0.1 and 1 µM, which align well for most of them with their IC_50_ values determined in the AS assay.

To investigate the specificity of the drugs for the intasome/nucleosome target, we tested them on integration assays performed on naked DNA. As reported in Fig. 4C, most of the drugs exhibited moderate inhibition effects on integration into plasmid pBSK-601-Zeo DNA, and the mitoxanthrone and tamoxifen compounds remained inefficient in this assay. Integration into naked biotin-601 immobilized DNA using similar amounts of DNA than in Fig. 4A and B confirmed that the inhibitory effect of the selected drugs was more important on assembled MNs than on the cognate naked DNA. Indeed, a comparison of the effect of the drugs on nucleosomal and naked 601 DNA (Fig. 4B and D) showed that while the IC_50_ values of the drugs on MN were between 0.1 and 1 µM, the IC_50_ values measured on naked DNA were all above 5–10 µM, suggesting a more specific effect of the drugs on nucleosomal DNA. The more pronounced effect of the drugs observed on the nucleosome led us to further investigate their possible action mechanism related to either the specific nucleosomal DNA structure or the histone octamer assembly.

### Effect of the chemical modulation of nucleosomal DNA topology and nucleosome stability by doxorubicin derivates on *in vitro* PFV integration

**Fig 5:**
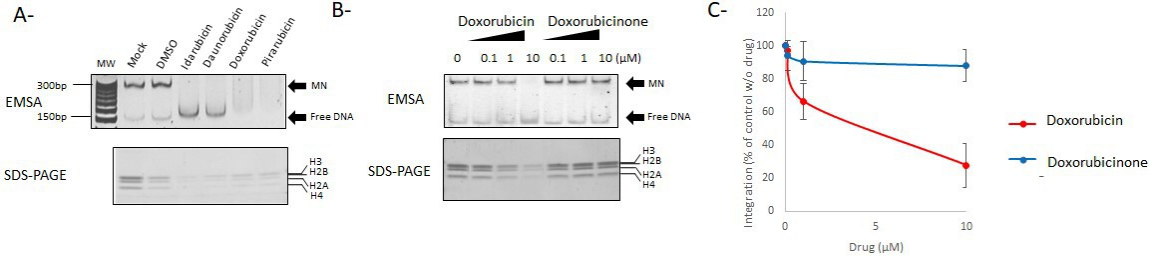
Action mechanism of doxorubicin derivates on nucleosomal integration. The doxorubicin derivates were tested in a histone eviction assay (**A**) using an effective concentration of 10 µM. Nucleosome structure was checked by electro-mobility shift assay (EMSA) and histone content analysis by instant blue-stained SDS-PAGE. Doxorubicin and its a-glycan form (doxorubicinone) have been compared in a histone eviction assay using increasing concentrations of drugs (**B**). The native MN and dissociated naked DNA positions in a SybrSafe-stained native 8% polyacrylamide gel are reported. Doxorubicin and doxorubicinone have been then tested on PFV concerted integration onto nucleosome using the increasing concentration of drugs as performed in Fig. 4. Data are reported as quantification of three independent experiments reported as mean ± SD in (**C**).

Based on the previous results, we assume that the drugs affected the target DNA with a preference for the nucleosomal structure. All the best-selected compounds were anthracycline enantiomers of doxorubicin known as DNA intercalating drugs (see their chemical structures in SI3). Intriguingly, no other DNA intercalating drugs included in the library, such as cisplatin, thalidomide, actinomycin D, and bleomycin, were selected, suggesting a specific inhibition mode of the selected doxorubicin derivates. The reported mechanism of action for anthracycline derivates, especially doxorubicin, is binding to DNA intercalated with base pairs, leading to specific binding of the molecules to guanine (as reported extensively in literature). Furthermore, the amino sugar group present in these compounds has also been reported to compete for space with the H4-arginine residues in the nucleosome that may lead to its dissociation (17). Based on their stronger inhibitory effect on MN integration and their DNA intercalating properties, we speculated that the integration inhibition may be due to possible binding to the nucleosomal DNA inducing destabilization of the MN structure. The possible destabilization effect of doxorubicin and its derivative on the MN structure has been further investigated using a previously described histone eviction assay (17). As shown in Fig. 5A, idarubicin and daunorubicin both induced a strong displacement of histones from the MN, leading to full dissociation of the MN and the releasing of free naked DNA. Doxorubicin and pirarubicin also induced a full shift of the nucleosomal DNA in addition to a strong MN dissociation. To better understand the link between the nucleosome disruption property of the doxorubicin derivate compounds and their integration inhibition property, we took advantage of the previous description of doxorubicinone as an aglycan form of doxorubicin that fails to evict histones from bound nucleosome (17). A histone eviction assay performed with doxorubicin and its aglycan form confirmed that doxorubicinone could not dissociate the MN in contrast to doxorubicin (Fig. 5B). As reported in Fig. 5C, doxorubicinone was also inefficient in inhibiting the *in vitro* integration catalyzed by the PFV intasome onto MNs. The correlation observed between the MN dissociation capability of these drugs and the integration inhibition strongly suggested that their inhibition mechanism was related to the doxorubicin-induced change in nucleosome structure leading to the competition of the amino sugar group of doxorubicin for space with the H4 arginine residue in the DNA minor groove, as previously predicted (17).

### Computational analysis of the effect of doxorubicin on intasome/nucleosome stability

To better determine the mechanism of doxorubicin inhibition and the capability of the drug to evict histones, even in a highly stable 601 nucleosome, its interaction with the nucleosome was first modeled using a 300-ns MD simulation as reported in Materials and Methods. As a control, the interaction of the nucleosome with doxorubicinone was also modeled. The doxorubicinone molecule only differs from doxorubicin in the lack of the glycan group, a group which has been reported to be important in the interaction of doxorubicin with the nucleosome.

**Fig 6:**
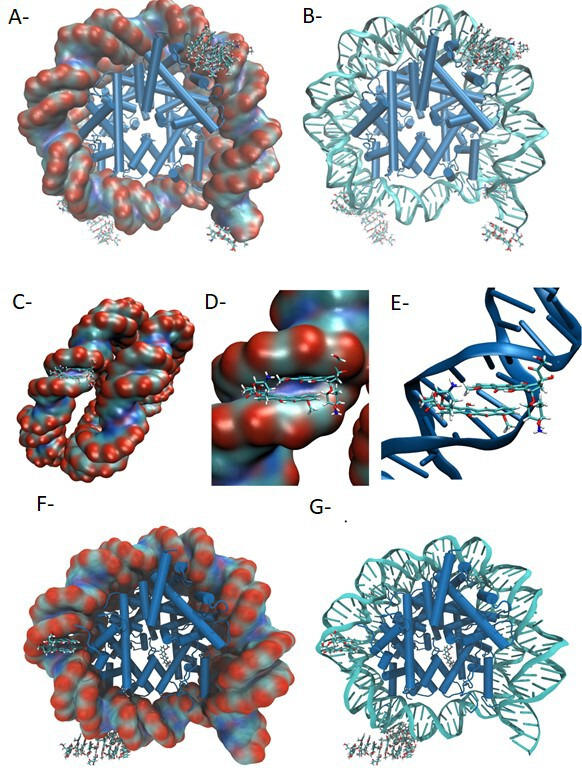
Modelization of the doxorubicin association with the nucleosome. The figure shows the structure of the nucleosome illustrating the overall distribution of the doxorubicin molecules after 300 ns of MD simulation. (**A**) Representation in the surface of DNA; (**B**) representation in the cartoon of DNA. In both representations, the histones are represented in cartoon and the doxorubicin molecules are represented in licorice. Water molecules were omitted for clarity. The representation of one of the most common doxorubicin-binding modes observed through the 300 ns of MD simulation, with two doxorubicin molecules adopting a planar conformation and intercalating between the two DNA helices, is reported in (**C**) as a surface view of DNA, (**D**) as the detail of the binding surface, and (**E**) as a cartoon representation of DNA. In all representations, the histones and water molecules are omitted for clarity and the doxorubicin molecules are represented in licorice. Modelization of the doxorubicinone association with the nucleosome was also performed in (**F**) and (**G**). The figure shows the structure of the nucleosome illustrating the overall distribution of the doxorubicinone molecules after 300 ns of MD simulation. (**F**) Representation in the surface of DNA; (**G**) representation in the cartoon of DNA. In both representations, the histones are represented in cartoon and the doxorubicinone molecules are represented in licorice. Water molecules were omitted for clarity.

Figure 6A represents the dominant conformation adopted by the doxorubicin molecules in the last 50 ns of the MD simulation. The simulation shows that the doxorubicin molecules interact with the nucleosome mainly at the DNA chains. Interestingly, while the initial MD conformation generated with packmol places the doxorubicin molecules randomly and uniformly distributed along the DNA surface, after the initial 20–50 ns of MD simulation, the doxorubicin molecules tend to pack together and crowd, intercalating between the DNA chains. As evidenced in Fig. 6A and B, these interactions typically involve two or more doxorubicin molecules, and once formed, they are very stable. SI5 presents the root-mean-square deviation (RMSD) of each of the doxorubicin molecules as calculated along the 300 ns of MD simulation. The results show that the conformations adopted by each doxorubicin molecule are quite stable once formed. Figure 6C through E shows one of the typical stable interaction modes observed from the MD simulation for the DNA-doxorubicin interaction. It involves an intercalation associated with two parallel doxorubicin molecules placed between the two DNA chains. As illustrated also in Fig. 6A and B, once formed, this binding mode is very stable and is maintained throughout the remainder of the simulation. On the other hand, even in later stages of the simulation, while the doxorubicinone also tend to pack together, only a few molecules interact with the DNA, as illustrated in Fig. 6F and G. In fact, while 15 of the 20 doxorubicin molecules remain in the vicinity of the nucleosome (75%), only six doxorubicinone molecules perform any direct interaction with the DNA or the histones (30%). SI5-B presents the RMSD of each doxorubicinone molecule.

The MM-GBSA calculation was then performed to obtain further information about the intasome/nucleosome dissociation effect of the selected doxorubicin drug. For this purpose, histone-DNA-binding free energy was estimated using MM-GBSA, and analyses of the RMSD and RMSF of the 300 ns MD simulations on the systems were performed with and without doxorubicin.

We first analyzed the impact of doxorubicin binding on nucleosome energetic stability. The protein-DNA-binding free energy ∆*G*_bind_ was estimated by MM-GBSA calculation. Values of the individual components are presented in Table 1, including the gas-phase electrostatic (∆*E*_ELE_) and van der Waals (∆*E*_VDW_) interaction energies, the polar solvation energy (∆*G*_GB_) calculated by using the generalized-born (GB) model, and the nonpolar solvation energy (∆*G*_Surf_). ∆*G*_Polar_ is written as the sum of the polar energy terms, which include ∆*E*_ELE_ and ∆*G*_GB_, while ∆*G*_Non-polar_ represents the sum of the nonpolar-based energy terms, which include ∆*E*_VDW_ and ∆*G*_Surf_. As reported in Table 1, the protein-DNA-binding free energy of −970.9 kcal/mol in the absence of any molecule dropped to a less stable binding free energy of −937.8 kcal/mol upon the addition of doxorubicin, leading to a predicted decrease in stability of 33.1 kcal/mol (3.4%). Analysis of the different individual contributions to the binding free energy, as calculated using the MM-GBSA method, showed that this difference arose from a decrease in both the polar (15.5 kcal/mol) and nonpolar components (17.5 kcal/mol), i.e., it has a 47% polar/53% nonpolar contribution. More detailed analysis of ∆∆*E*_ELE_, ∆∆*E*_VDW_, ∆∆*G*_GB_, and ∆∆*G*_Surf_ upon doxorubicin-binding unambiguously showed that the doxorubicin interaction decreased the direct electrostatic and van der Waals interactions between protein and DNA. Consequently, based on these calculations, doxorubicin binding to MN should lead to a less stable structure. As for doxorubicinone, the protein-DNA-binding free energy also dropped from −970.9 to −945.6 kcal/mol. However, as expected, this decrease was smaller than what was observed in the simulation with doxorubicin (decrease of 2.6% for doxorubicinone and 3.4% of doxorubicin). This indicates that doxorubicinone is not capable of affecting the stability of the MN in the same extent as doxorubicin.

**TABLE 1:** Nucleosome histone-DNA-binding free energies estimated by MM-GBSA in the absence and presence of doxorubicin or doxorubicinone^*a*^

Model	∆*G*_bind_ (kcal/mol)	∆*E*_ELE_ (kcal/mol)	∆*E*_VDW_ (kcal/mol)	∆*G*_GB_ (kcal/mol)	∆*G*_Surf_ (kcal/mol)	∆*G*_Polar_ (kcal/mol)	∆*G*_Non-polar_ (kcal/mol)
Without molecules	−970.9 ± 1.0	−163,522.1 ± 16.7	−764.3 ± 0.5	163,422.2 ± 16.4	−106.6 ± 0.1	−99.9	−870.9
With doxorubicinone	−945.6 ± 0.9	−163,075.8 ± 16.8	−783.0 ± 0.6	163,022.0 ± 16.2	−108.7 ± 0.1	−53.9	−891.87
Variation	+25.3	+446.3	−18.7	−400.2	−2.1	+46.0	−20.8
With doxorubicin	−937.8 ± 0.9	−163,161.3 ± 17.6	−748.6 ± 0.6	163,076.9 ± 17.3	−104.8 ± 0.1	−84.4	−853.4
Variation	+33.1	+360.8	+15.7	−345.3	+1.8	+15.5	+17.5

^*a*^ All values in kcal/mol.

We then analyzed the impact of doxorubicin binding on nucleosome structural stability. Table 2 presents the average RMSD of the last 200 ns of the MD simulations performed in the absence and presence of doxorubicin, as well as the presence of doxorubicinone, in comparison with the nucleosome X-ray structure. The results show that the average RMSD of the backbone atoms in the nucleosome is larger in the simulation performed in the presence of doxorubicin (2.77 Å) than in the simulation performed in the absence of doxorubicin (2.67 Å), illustrating that the presence of doxorubicin induces a structural change. When doxorubicinone is present, the average RMSD of the nucleosome (2.67 Å) is identical to when there are no additional molecules in the system (2.67 Å). Analyzing the behavior of the protein and DNA components of the nucleosome, it can be observed that this effect is stronger in DNA than in histones. In fact, DNA exhibits a larger RMSD change with doxorubicin addition (from 3.33 to 3.58 Å) than the histones do (from 1.43 to 1.48 Å). This was confirmed when regarding the RMSD change over time for both all simulations (SI4) showing the same average tendency described in Table 1: the RMSD is larger for the simulation in the presence of doxorubicin than it is for the simulations in the absence of doxorubicin, and the RMSD change with doxorubicin addition is larger for DNA than it is for the histones. As for doxorubicinone addition, the RMSD values for both the histones and the DNA are similar to what is observed when there are no added molecules to the system. These results indicate that the lack of the glycan group greatly affects the ability of doxorubicinone to interact with the nucleosome. SI4 also demonstrates that both simulations are well equilibrated after the initial 50 ns.

**TABLE 2:** Average RMSD of the last 200 ns of the MD simulations in comparison with the initial nucleosome structure in the absence of any molecule and in the presence of doxorubicine and doxorubicinone

Model	Average RMSD nucleosome (Å)	Average RMSD histones (Å)	Average RMSD DNA (Å)
Without molecules	2.67 ± 0.13	1.43 ± 0.07	3.33 ± 0.18
With doxorubicinone	2.67 ± 0.18	1.46 ± 0.10	3.29 ± 0.26
With doxorubicin	2.77 ± 0.14	1.48 ± 0.08	3.58 ± 0.22

The impact of doxorubicin binding on nucleosome structural flexibility was then investigated. Table 3 presents the average root-mean-square fluctuation (RMSF) of the backbone atoms in the nucleosome, illustrating the positional variability/flexibility of the atoms in the nucleosome in the presence and absence of doxorubicin and doxorubicinone and in the absence of any molecule. The results show that the addition of doxorubicin alters the flexibility of the nucleosome. On average, doxorubicin induces a slight increase in the flexibility of the nucleosome residues (1.12 vs 1.09 Å). This effect is more significant for DNA (1.62 Å with doxorubicin vs 1.51 Å without doxorubicin) than it is for histones. As expected, the addition of doxorubicinone does not lead to any increase in the flexibility of the nucleosome (1.07 vs 1.09 Å). SI5 illustrates the RMSF for the different amino acid and nucleotide positions along the histone and DNA chains in the nucleosome in the absence and presence of doxorubicin and doxorubicinone and in the absence of any molecule. The results show a similar profile in terms of relative flexibility for both models. In general, the most flexible amino acid positions and DNA positions are the same, independent of the presence of any molecule. However, the simulation in the presence of doxorubicin shows an increase in flexibility, which is particularly noticeable for the DNA portion of the nucleosome. These changes are not evenly distributed but are stronger along specific positions in the nucleosome. SI6 represents the individual change in RMSF with the addition of doxorubicin. The results confirm the existence of general light flexibility variations along the histone residues, with more significant increases along the amino acid positions/histones 127A-135A, 29D-47D, 134E-25F, and 30H-32H. Decreases in flexibility were also observed along positions 19B-22B, 91F-96F, and 97G-100G. RMSF changes along the DNA positions upon doxorubicin interaction were much more dramatic and particularly evident among positions –72/–57, –52/–22, and 67/72 in chain I and –72/–63 and +31/+68 in chain J. These regions are more affected by the interaction with doxorubicin molecules. On the other hand, the presence of doxorubicinone does not lead to any significant increase of flexibility. SI6 demonstrates that both DNA and histone RMSF values in the simulations with doxorubicinone are lower when compared to doxorubicin and very similar when compared to the simulation without any added molecules. Once again, this indicates the importance of the glycan group of the doxorubicin molecule.

**TABLE 3:** Average RMSF of the two nucleosomes and the histone and DNA components in the absence and presence of doxorubicin and doxorubicinone

Model	Average RMSF nucleosome (Å)	Average RMSF histones (Å)	Average RMSF DNA (Å)
Without molecules	1.09 ± 0.46	0.92 ± 0.39	1.51 ± 0.35
With doxorubicinone	1.07 ± 0.45	0.90 ± 0.35	1.52 ± 0.36
With doxorubicin	1.12 ± 0.54	0.93 ± 0.42	1.62 ± 0.51

### Selection of drugs targeting the protein/histone interaction as inhibitors of PFV integration

**Fig 7:**
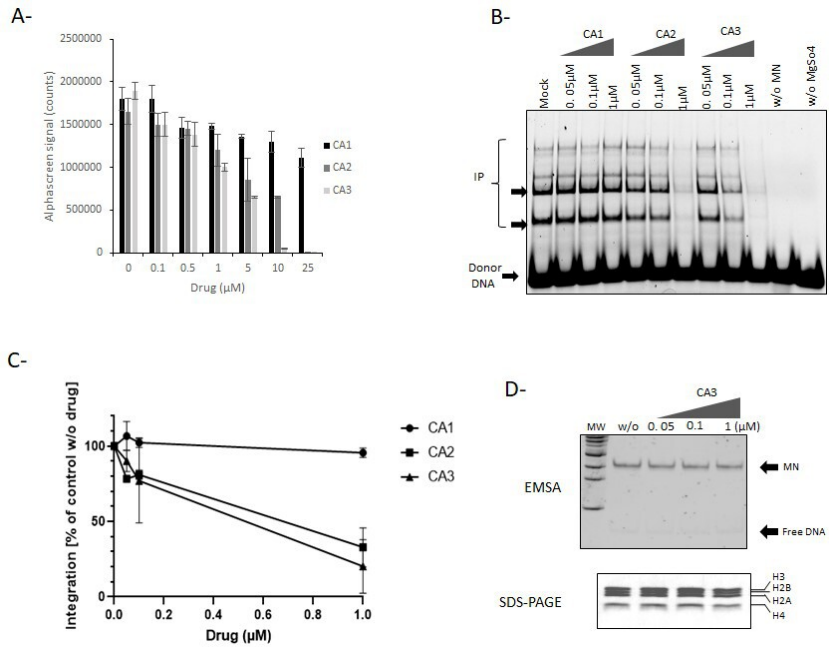
Effect of calixarenes compounds on PFV intasome/nucleosome complex formation and *in vitro* integration. CA1, CA2, and CA3 molecules have been tested on the PFV intasome/nucleosome complex using the AlphaLISA technology as setup in this work (**A**). The drugs were then assayed in a typical concerted integration using FITC-PFV intasome and nucleosome, and the integration products were monitored on 8% native polyacrylamide gel (**B**). The results are reported in (**C**) as the mean of three independent integration experiment ± SD. IP, integration products. The CA3 drug was tested in a histone eviction assay (**D**) using the increasing concentration of the drug. Nucleosome structure was checked by electro-mobility shift assay (EMSA) and histone content analysis by instant blue-stained SDS-PAGE.

In addition to histone/DNA interactions destabilized by doxorubicin drugs binding to the nucleosome, protein/histone interactions should also constitute important components of retroviral intasome/nucleosome stability, as previously suggested (10, 12, 13). Tetrasuflonated calix[4]arene molecules have been previously reported and specifically bind to histone tails with submicromolar affinity and strong specificity [(18) and Table 4]. Among these compounds, CA3 (see the chemical structure in SI7) showed the best histone-binding affinity and was proposed to be effective for disrupting interactions between histone tails and effector proteins (18). These molecules were, thus, also included in the intasome/nucleosome AS assay to evaluate their capability to dissociate or block the formation of the PFV intasome/MN complex. As reported in Fig. 7A, among the calixarene drugs, the CA3 compounds were shown to prevent the formation of the intasome/MN complex in AS, leading to its complete dissociation at concentrations greater than 5 µM. CA2 and CA1 analogs were found to be less efficient in inhibiting the association, which aligns well with their lower histone affinity (see their previously measured Kd for histone tails in Table 4). No effect of the drugs was observed in counter select assay (see SI2).

**TABLE 4:** Comparison between the histone tails affinity and *in vitro* HIV-1 integration inhibition of CA molecules

CA molecule	Kd (µM) to histone tails (18)	Integration IC_50_ (µM)
CA1	17.8–20.4	>1
CA2	1.8	0.9
CA3	0.73–0.51	0.1–0.4

An *in vitro* concerted integration assay performed on MNs confirmed that CA3 inhibited vDNA insertion catalyzed by the PFV intasome (Fig. 7B). Comparison of the inhibitory effect of CA1, CA2, and CA3 on *in vitro* integration catalyzed by the PFV intasome also showed different efficiencies (Fig. 7C). Indeed, while CA3 showed a stronger inhibitory effect with IC_50_ ~100–400 nM, CA2 showed a lower inhibition capability (IC_50_ ~900 nM); CA1 was inefficient in our assay. Comparison with their effect on AS showed a similar ranking of the molecules with the CA3 being the most efficient in dissociating the intasome/nucleosome. However, the drugs were found more efficient in inhibiting integration than intasome/nucleosome association, suggesting that the molecule may allow keeping some DNA-intasome interactions that may be sufficient for residual AS signal, while the loss of histone interaction due to the drugs may have a higher impact on integration. This is supported by the intasome activation induced by histone tail-mediated structural changes as observed for HIV-1 (13). No effect of inhibitory compound CA3 was observed on nucleosome structure which is in agreement with the competitive effect of the drug with the intasome binding to the histone tails (Fig. 7D).

### Effect of the selected compounds on *in vitro* HIV-1 integration

**Fig 8:**
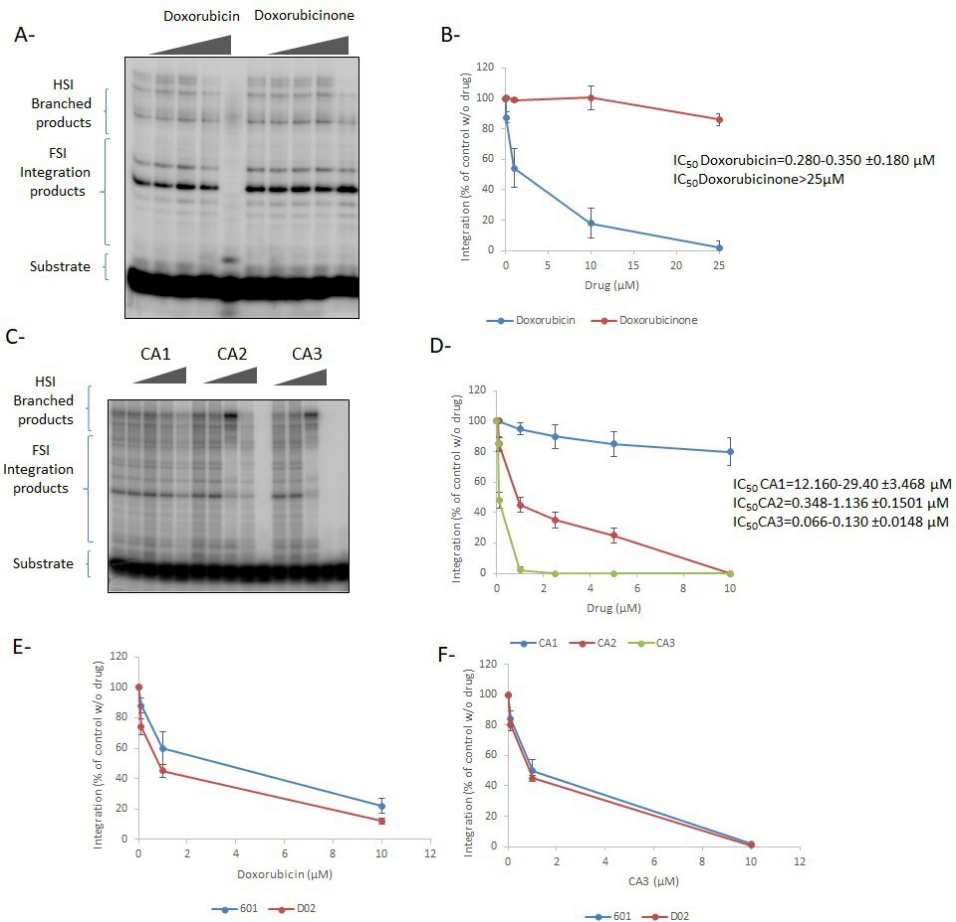
Effect of the selected drugs on *in vitro* HIV-1 integration. Doxorubicin /doxorubicinone (**A and B**) or CA1/2/3 (**C and D**) have been added to a typical *in vitro* concerted integration performed with preformed HIV-1 intasome, radiolabeled viral U5 end fragment, and mononucleosome. The integration products were monitored on 6–12% gradient polyacrylamide gel (left panels) and quantified (right panels). Inhibition assays were also performed using the DO2 nucleosome for doxorubicin (**E**) and CA3 (**F**). Data are reported as the mean of two to four independent experiments ± SD.

To better address their specificity, the best-selected drugs were further tested for HIV-1 integration using an *in vitro* integration assay and performed on MNs with preformed functional vDNA•IN•LEDGF/p75 intasome complexes as previously described (9). As reported in Fig. 8A and B, doxorubicin was also shown to inhibit the *in vitro* integration of HIV-1 with similar efficiency to that shown by the PFV model (IC_50_ ~300 nM). Again, the doxorubicinone derivative was found to be inefficient, strongly suggesting that the mechanism of action of doxorubicin was similar to that elucidated in the PFV model, i.e., nucleosome dissociation by histone eviction (see Fig. 5 and 6). Integration assays performed with the CA3 compound showed that it was also efficient in inhibiting HIV-1 *in vitro* integration (Fig. 8C). Comparison between the inhibition efficiency of CA1, CA2, and CA3 showed a good correlation between the histone tail-binding property of the drugs and their integration inhibition efficiency, with CA3 being the most efficient compound with an IC_50_ of approximately 100 nM (see the determination of the IC_50_ for all molecules in Fig. 8D). Based on their affinity for histone tails, the inhibition mechanism was assumed to be a competition between the intasome and these tails. This was confirmed by an integration inhibition assay performed on native MN, tailless MN, or naked DNA, showing that deleting the tails or using naked DNA increased the IC_50_ of the drug from 0.035 to 0.092 µM, 0.127 to 0.913, and 0.178 to 0.810 µM, respectively (SI7). These results confirmed that the main mechanism for the integration inhibition mediated by CA3 involves competition with intasome binding to the histone tails.

To investigate the possible specificity of the integration inhibition by the drugs toward the DNA target sequence, we performed additional inhibition assays using MN assembled onto the natural DO2 sequence. This MN has been previously reported to be a good substrate for retroviral integration (10). As reported in Fig. 8E and F, both doxorubicin and CA3 were as efficient in inhibiting the HIV-1 integration into DO2 nucleosome than onto the 601 nucleosome suggesting no significant DNA sequence specificity for the integration inhibition and underlining the inhibitory capability of both drugs onto natural nucleosomal sequence.

### Effect of the selected compounds on *in cellulo* HIV-1 replication

Next, we further address the efficiency of the drug in a cellular context. We first checked the cytotoxicity of the compounds in different cell lines to evaluate their possible use as antiviral agents. MTT cytotoxicity assays performed with the drugs selected using the AS screen of the OncoSET library, including the doxorubicin derivatives, showed that they were all cytotoxic in typical transformed or cancer cellular models within different concentration ranges (see SI8). This was expected since these compounds are all anticancer drugs that act by inducing the death of cancerous cells. In contrast, a poor effect was observed in noncancerous primary PBMCs. In contrast to doxorubicin derivatives, the CA molecules were shown to have little to no cytotoxicity in our assays in all the cell lines, including PMBCs (see SI8). Based on these data, both selected drugs were assayed for HIV-1 infection in primary PBMCs.

**Fig 9:**
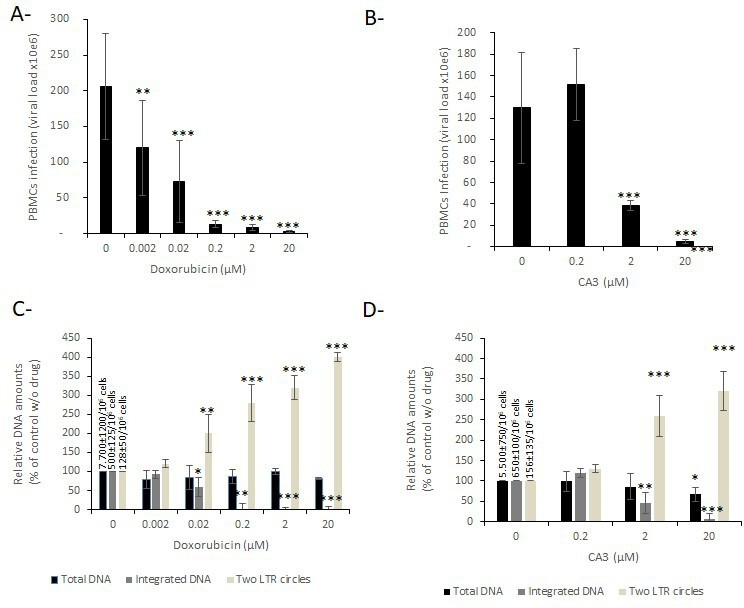
Effect of the doxorubicin and CA3 on HIV-1 replication. PBMCs cells have been infected with HIV-1 virus in the presence of the increasing concentration of drugs. Replication was quantified by HIV-1 RNA determination in cellular supernatant (**A and B**). The viral DNA copy number per 10^6^ cells has been reported in the figure. Viral DNA populations have been quantified by qPCR (**C and D**). Data are reported as the mean of two to four independent experiments ± SD. *P < 0.05, **P < 0.005, ***P < 0.001.

Infection assays performed with the clinical B subtype HIV-1 strain in PBMCs treated with doxorubicin showed a strong inhibition of replication by the drug, leading to an IC_50_ of 2–20 nM (Fig. 9A). The CA3 compound was also found to decrease viral replication with an IC_50_ of 1–2 µM (Fig. 9B). qPCR quantification of the total DNA at 20 h postinfection showed no effect of the doxorubicin, indicating that the drug does not affect the reverse transcription or the entry step (Fig. 9C). In contrast, integrated DNA was found to be decreased in doxorubicin-treated cells, while two-LTR circle amounts were increased (Fig. 9C). These data confirmed that doxorubicin inhibited the postnuclear integration steps. Quantification of the total DNA in infected cells treated with CA3 showed no effect of the drugs on room temperature (RT) at concentrations up to 2 µM, where a severe decrease in replication was observed (Fig. 9D). However, higher concentrations (20 µM) induced a slight decrease in total DNA synthesis, suggesting that under these concentrations, the drugs might affect reverse transcription or virus entry. The integration inhibition was confirmed by quantification of the integrated and unintegrated circular DNA showing the expected signature of integrated DNA increase accompanied by an increase in two-LTR circles.

The specific analysis of the effect of doxorubicin and CA3 on viral entry was performed by typical viral fusion quantification using BLaM assay. The data confirmed that both drugs did not affect this early entry step in contrast to the well-known T20 inhibitor (SI9). Further comparison with known RT and IN inhibitors (namely, efavirenz and dolutegravir; see SI10) confirmed that CA3 histone binder and doxorubicin target the integration in infected cells and may serve as tools for further dissecting the retroviral intasome/chromatin interaction and constitute candidates for further therapeutic developments.

### Conclusions

The retroviral intasome/nucleosome complex is the functional minimal entity responsible for the stable integration of the viral genome into the host chromatin. Structural data about this complex are available only for the PFV model (10). These data show that the intasome/nucleosome complex involves multiple types of interactions, including protein-protein and protein-DNA interactions within the nucleosome, the IN-IN oligomer, or the intasome-nucleosome complex (10). Chemical modulation of these interfaces is an original way to better understand the role of these interactions in the integration process as well as help the development of potential molecular tools or therapeutic agents. Both the understanding of the functional architecture of this complex and the search for new inhibitory strategies are required to monitor all these IN/nucleosome interfaces. To this end, we have developed an AlphaLISA-derived approach allowing us to recapitulate most of the interactions engaged in the retroviral intasome/nucleosome complex, including the IN/target DNA, IN/vDNA, IN/IN, and IN/histone bonds. In addition to providing an experimental model for further depicting the role of these interactions, our approach was used to select new molecules that dissociate the functional complex by targeting each or all of these interfaces.

Since chromatin is a target for anticancer therapy, the NIH OncoSET library was first screened. Among the 133 drugs in the OncoSET database, four were selected as significant inhibitors of both the AS intasome/MN interaction signal and *in vitro* integration. Most of the selected drugs were anthracycline derivates, including doxorubicin, and are known as DNA intercalating agents. Interestingly, inhibition of HIV-1 IN activity by doxorubicin has been previously reported in strand transfer assays performed onto naked DNAs (19). The inhibition of this partial strand-transfer activity onto naked short DNAs has been reported with IC_50_ values between 1 and 10 µM which parallel finely what we observed using longer naked plasmid DNA and PFV intasome (Fig. 4). However, the higher efficiency of the drugs reported in this work in inhibiting integration into nucleosome for both PFV and HIV-1 suggests a new mechanism of action for this molecule acting specifically on the physiological nucleosomal DNA substrate with IC_50_< 1µM. Interestingly, not all the intercalating agents represented in the OncoSET library, such as actinomycin D, showed AS signal inhibition effects. More strikingly, among the anthracyclines in the OncoSET database, not all were selected. For instance, epirubicin was not selected as an intasome/nucleosome modulator, in contrast to doxorubicin, idarubicin, pidorubicin, and daunoribicin, suggesting a specific action mechanism for these anthracycline derivatives, leading us to further investigate the nucleosome dissociation property of doxorubicin as a mechanism for the reported inhibition. The nucleosome dissociation property of this molecule has been previously reported to be due to the competition of the amino sugar group of the drug for space with the H4-arginine residue in the DNA minor groove since this H4-DNA interaction stabilizes the nucleosome structure (20). Both computational and biochemical analyses of the mechanism of action of the drug confirmed that the main process leading to integration inhibition was the dissociation of the nucleosome structure by the histone eviction induced by the competition for the space with the H4-arginine residues in the nucleosome mediated by the amino sugar group present in these compounds. The lack of effect observed with other strong DNA intercalating agents, such as actinomycin D, also suggested that the dissociation of the nucleosome induced by the binding of drugs to DNA is the prerequisite for efficient integration inhibition of this substrate due to the destabilization of the MN structure by affecting the nucleosomal DNA topology. To better understand how doxorubicin could dissociate the 601 nucleosome despite its high stability, we further performed computational MM-GBSA analyses. These analyses confirmed that the binding of doxorubicin on the nucleosomal DNA induces physical constraints at both the DNA and protein levels, leading to the destabilization of the complex. In contrast, the lack of glycan group on the doxorubicinone greatly decreased the ability of the molecule to interact with the nucleosome in addition to decrease its capability to inhibit integration. Furthermore, the simulations revealed that several doxorubicin molecules can bind one nucleosome inducing cumulative destabilization of the structure in the different binding sites that may lead to the total dissociation of the nucleosome. This hypothesis is strengthened by the fact that the molecules were found to tend to pack together and crowd probably increasing their dissociation capability. Taken together, these data suggest a new integration inhibition process specifically targeting the physiological nucleosome substrate by anthracycline derivates. The results also confirm the importance of the fully assembled nucleosome for efficient integration, shedding light on previous observations showing that the remodeling process favors HIV-1 integration (7, 21). Indeed, altogether, these data suggest that enhancement of integration may require the remodeling of neighboring chromatin, allowing the intasome to gain access to a fully native-targeted nucleosome or polynucleosome at the insertion site. Integration inhibition observed in HIV-1-infected primary cells confirms that these interactions are also important in the physiological complex.

With the same methods of probing the protein/DNA interfaces, we also wanted to evaluate the IN/histone interaction importance by chemical targeting using histone binders as previously reported calixarene molecules (18). The AlphaLISA approach led us to show the inhibition of the intasome/nucleosome interaction by the CA3 drug. *In vitro* integration assays further demonstrated that this drug was able to inhibit nucleosomal integration without affecting the nucleosome structure. An assay on other retroviral integration systems, such as HIV-1, confirmed that the selected drugs could also be efficient in inhibiting this integration process *in vitro,* further validating the approach for selecting drugs aimed at targets of potential therapeutic interest. Comparison of the effect of the CA3 drug and chemical analogs with less affinity for histone tails, in addition to assays using tailless nucleosomes, confirmed the histone-dependent inhibition mechanism of the drug. These results validated the chemical dissociation of the IN/histone tail interaction as a new retroviral inhibition mechanism. These results parallel those of previously published data showing that calixarene compounds could inhibit histone binding by histone readers both *in vitro* and *in cellulo* (22, 23). In this line, our work paves the way for the possible development of calixarenes derivates specific for some modifier histone tails possibly recognized by retroviral intasomes (such as the H3K36me3 modification assumed to be associated with LEDGF/p75, the histone H4 tail bound by HIV-1, or H4ac associated with BET proteins participating to the chromatin anchoring of gammaretroviral intasomes) (24, 25). Our results also confirm the importance of intasome/histone interactions for efficient HIV-1 integration, as previously suggested (12, 13) and demonstrated for the PFV model (10).

In addition to serving as interesting tools for dissecting the molecular processes involved in the functional anchoring of the intasome to the nucleosome, we wondered whether the selected compounds could also be the foundation of new therapeutic approaches. To this end, we further studied the inhibitory capability of the selected drugs on the HIV-1 model of direct therapeutic interest, focusing on doxorubicin and CA3. Both doxorubicin and CA3 compounds showed poor cytotoxicity in primary PBMCs up to 38 µM, which allowed us to investigate their effect on viral infectivity. The data indicated that the two drugs were able to inhibit HIV-1 infectivity with IC_50_ values between 2 nM and 1–2 µM. Quantification of the viral integrated and unintegrated DNA populations confirmed that the drugs were able to specifically block the integration step without affecting the entry step as confirmed by BLaM assay.

Additional work will be required for optimizing the inhibitory property of the drugs as well as improving the cellular tolerance of them. While the exact inhibition mechanism in the infected cells remains to be fully described, our data show that the selected drugs are good candidates for new antiviral lead compounds and provide a new antiviral precept that may pave the way for further developments of such strategies targeting the integration step. Recent work showed that purified PIC may display distinct behavior that isolated intasome especially in their preference for histone-modified nucleosomes or naked DNA (26). Thus, testing the selected compounds on these purified complexes may provide additional clues in their action mechanism. However, the currently selected drug may serve for further structure-activity (SAR) studies to both improve their inhibition efficiency and specificity and decrease possible cellular toxicity. To this end, our study provided some information for these future improvements. Indeed, epirubicin is a stereoisomer in which the hydroxyl group of doxorubicin is inverted at position 4′ and has a similar mechanism of action as that of doxorubicin, while the compound is inefficient in inhibiting the intasome/nucleosome complex. This may underline the importance of position 4′ in the inhibition mechanism. The inefficiency of valrubicin may also be taken into account for SAR studies. The biochemical data as well as the *in silico* GBSA modeling provided in our work will also serve as a basis for such improvement. The validation of the AlphaLISA reported here also opens the way for broader screening using larger libraries that should allow the identification of additional compounds modulating the intasome/nucleosome interfaces important for the integration process. Indeed, no drugs targeting the intasome alone have been selected, probably due to the nature of the selected library encompassing mainly drugs targeting the chromatin components. An additional library may circumvent this issue and lead to the selection of additional inhibition processes.

In addition to identifying compounds interfering with intasome/nucleosome binding, our data confirm that the AS approach reported here constitutes a robust model for selecting drugs able to modulate the intasome/nucleosome interfaces in several retroviral models, including those of therapeutic interest. Both anthracycline derivatives and the histone binder CA3 selected in this system using the PFV model were active in inhibiting *in vitro* HIV-1 integration, confirming that the PFV intasome/nucleosome AlphaLISA approach is suitable for selecting potential anti-HIV compounds. Data obtained with the nucleosome assembled onto natural DO2 sequence confirm that the 601 nucleosome used in our initial screen is a relevant model for such studies. However, we cannot rule out that some features present in physiological chromatin may not be recapitulated properly in our system. The use of additional nucleosome structures, or more complex polynucleosomes, in the AS could also be an alternative to select molecules targeting more physiological complexes.

However, our results provide evidence that AlphaLISA is a technology also suitable for bimolecular inhibitor screening assays using large complexes such as intasomes (several hundreds of kDa) and nucleosomes (~200 kDa). This approach has the additional advantage of recapitulating all the interfaces engaged in these large nucleocomplexes, allowing the selection of molecules that may target all these interfaces, in contrast to approaches based on minimal partners. In our study, the AS approach particularly allowed us to identify drugs targeting either the MN tail or the DNA. The developed model and the selected drugs will help in better dissecting the molecular interactions within the intasome/nucleosome functional complex. In particular, this first monitoring of the retroviral/intasome complex paves the way for multiple applications, including the analysis of the influence of cellular partners and the study of additional retroviral intasomes, allowing us to determine the possible specific interfaces previously suggested (8, 9, 27). Our work also provides the technical basis for larger-scale screening of drugs specifically targeting these functional nucleocomplexes or additional nucleosome-partner complexes.

## MATERIALS AND METHODS

### Proteins

IN of PFV has been purified and the intasome assembled as described in reference (10). Briefly, PFV IN and its cognate vDNA were mixed, and assembled intasomes were purified by size exclusion chromatography. Elution profile of the intasome used in this study is shown in Fig. 1A and, concordantly with published works, the PFV intasome, which is composed of a tetramer of IN, eluted around 11 mL. The HIV-1 IN was expressed in *Escherichia coli* (Rosetta), and the cells were lysed in buffer containing 50 mM Hepes pH 7.5, 5 mM EDTA, 1 mM DTT, and 1 mM PMSF. The lysate was centrifuged and IN extracted from the pellet in buffer containing 1 M NaCl, 50 mM Hepes pH 7.5, 1 mM EDTA, 1 mM DTT, and 7 mM CHAPS. The protein was then purified on butyl column equilibrated with 50 mM Hepes pH 7.5, 200 mM NaCl, 1 M ammonium sulfate, 100 mM EDTA, 1 mM DTT, 7 mM CHAPS, and 10% glycerol. The protein was further purified on heparin column equilibrated with 50 mM Hepes pH 7.5, 200 mM NaCl, 100 mM EDTA, 1 mM DTT, 7 mM CHAPS, and 10% glycerol. LEDGF/p75 was expressed in PC2 bacteria, and the cells were lysed in lysis buffer containing 20 mM Tris-HCl pH 7.5, 1 M NaCl, 1 mM PMSF added lysozyme, and protease inhibitors. The protein was purified by nickel-affinity chromatography, and the His-tag was removed with 3C protease, 4°C over night. After dilution down to 150 mM NaCl, the protein was further purified on SP column equilibrated with 25 mM Tris pH 7.5, 150 mM NaCl (gradient from 150 mM to 1 M NaCl), DTT was added to 2 mM final, and then, the protein was concentrated for Gel filtration. Gel filtration was performed on a superdex 200 column (GE Healthcare) equilibrated with 25 mM Tris-HCl pH 7.5 and 500 mM NaCl. Two mM DTT were added to the eluted protein that was then concentrated to about 10 mg/mL.

### Drugs

The drugs library used in this work is a 133 FDA-approved drugs library kindly provided from NCI/DTP Approved Oncology Drugs Plated Set (AODVIII,https://dtp.cancer.gov/organization/dscb/obtaining/available_plates.htm). This library is derived from the Approved Oncology Drugs Set. The selected compounds were then purchased from various private companies. The CA compounds were synthetized as previously described (18).

### Nucleosome assembly and eviction assay

MNs were assembled as previously described for chromatin assembly (7, 8). Briefly, 5 µg of biotinylated 147 bp Widom fragment (TEBU-bio) were mixed with an excess of 10 µg of human native recombinant octamers produced in *E. coli* [purchased from the “Histone Source” Protein Expression and Purification (PEP) facility from the Colorado State University, https://histonesource-colostate.nbsstore.net] in Tris-HCl pH 7.7 and 2 M NaCl in 100 µl final volume. Salt dialysis was then performed to decrease the salt concentration to 0 using slide-A-Lyzer MINI dialysis device, 7 k MWCO (Fisher Scientific). Assembly was checked by electro-mobility shift assay (EMSA) on 8% native PAGE stained with SYBR safe and SDS-PAGE stained with instant blue. Eviction assays of the assembled nucleosome were incubated with increasing concentrations of drugs and then analyzed by EMSA or SDS-PAGE of the supernatant after their capture using streptavidin beads (MyONE T1 invitrogen). Naked DNA substrates (pBSK-601-Zeo vector plasmid containing 601 Widom repetitions or 147 bp 601 DNA fragment) used for comparative integration assays were treated as for nucleosome assembly.

### Integration assays using the intasomes

Integration assays using purified assembled intasomes were performed as followed: either 100 ng of naked DNA or 100 ng of DNA assembled on 100 ng of histone octamers (24 nM final concentration) was incubated with 30 nM final concentration of purified intasome in 100 mM NaCl, 20 mM BTP pH 7, 12.5 mM MgSO_4_ (for PFV intasome), or in 20 mM HEPES pH 7, 7.5% DMSO, 8% PEG, 10 mM MgCl_2_, 20 µM ZnCl_2_, 100 mM NaCl, 5 mM DTT final concentration (for HIV-1 intasome) in a final volume of 40 µL. The mix was then incubated at 37°C for 15 min and 1 h, respectively. Then, reaction was stopped by the addition of 5.5 µL of a mix containing 5% SDS and 0.25 M EDTA and deproteinized with proteinase K (Promega) for 1 h at 37°C. Nucleic acids were then precipitated with 150 µL of ethanol overnight at −20°C. Samples were then spun at top speed for 1 h at 4°C, and the pellets were dried and then resuspended with DNA loading buffer. Integration products were separated on an 8% native polyacrylamide gel.

### AlphaLISA

AlphaLISA is a technology particularly suitable for bimolecular inhibitor screening assays using protein-protein interactions with purified recombinant proteins. The AlphaLISA assay development was performed in 96-well 12 area Alphaplate (reference 6052340, PerkinElmer, Waltham, MA, USA) with a final reaction volume of 40 µL. Cross-titration experiments of biotinylated MN (0–50 nM) against DIG-intasome (0–100 nM) were carried out to establish optimal assay concentrations for the binding assay. Ten microliters of each protein were diluted in the binding buffer 50 mM BisTris-propane pH 7, 100 mM NaCl, 0.1% (v/v) Tween-20, 0.1% (w/v) BSA, and 1 mM dithiothreitol. The plate was incubated at RT for 2 h in rotation. Ten microliters of anti-DIG acceptor beads (PerkinElmer, reference AL113) were then added, and after 1 h of incubation at RT with rotation, 10 µL of streptavidin donor-acceptor beads (Perkin Elmer, reference 6760002) was mixed to the wells. This established a final concentration of 20 µg/mL for each bead. The plate was then incubated for 1 h at RT in the dark before the AlphaLISA signal was detected using an EnSpire Multimode Plate Reader (PerkinElmer). Negative control with binding buffer or only with one of the complexes was used to control the assay quality. Data were analyzed with GraphPad Prism 5.01 software. To evaluate tolerance for DMSO, the assay was performed as described above with an addition of 0.15–20% (v/v) of DMSO during the binding step. For the competition experiment, increasing concentrations of untagged intasome were titrated out in the 1-nM DIG-intasome/biotinylated nucleosome interaction assay.

For the AlphaLISA screening assay with the NIH OncoSET library, the binding conditions were optimized for use in 384-well plate (Optiplate, reference 6007290, PerkinElmer) with the same concentrations for complexes and beads in a final reaction volume of 20.5 µL (2.5% DMSO final concentration). The screening was first carried out with 25 µM of each compound pre-incubated for 1 h with 1 nM intasome/nucleosome complexes. Compounds were selected as inducing at least 50% decrease of the AlphaLISA signal control condition, and their inhibitory effect was further tested using dose-response curves. For compounds validation, counter select assay was performed using a short DNA fragment carrying a biotin and a DIG tag on each side. The AlphaLISA signal was monitoring using increasing concentrations of the double-tagged DNA, and the selected compounds were tested in optimized conditions at 10 µM. Any compound that causes decreased signal means that it interferes with AlphaLISA readout, and therefore, it is not relevant to this assay.

### MD simulation set-up and parametrization

To evaluate the impact of the presence of doxorubicin and doxorubicinone on MN stability, two models were prepared: one model of the nucleosome complex; another including the nucleosome complex plus 20 doxorubicin molecules; a final model including the nucleosome complex and 20 doxorubicinone molecules.

The model for the nucleosome was prepared from the 5MLU structure (28), available in the Protein Databank (29), with a resolution of 2.80 Å. Protonation of all the amino acid residues was predicted using Propka version 3.0 at pH 7.0 (30). For the model with the doxorubicin and doxorubicinone molecules, packmol (31) was used to place and randomly distribute 20 doxorubicin and doxorubicinone molecules (one for each seven base pairs) around the volume defined by the two planes aligned with the enveloping DNA chains of the nucleosome.

All systems were further prepared for molecular dynamics simulations using the AMBER18 software package and Xleap, using the ff14SB for the amino acid residues (32), the OL15 force field for DNA (33), and the General Amber Force Field (GAFF) for doxorubicin (34). Charges on the systems were neutralized through the addition of counter-ions, and the system was placed in with TIP3P water boxes with a minimum distance of 12 Å between the nucleosome surface and the side of the box, using the LEAP module of AMBER.

Doxorubicin and doxorubicinone were parameterized with Antechamber using GAFF with RESP charges determined at HF/6–31G(d) using Gaussian16.

All systems submitted to four consecutive minimizations stages to remove clashes prior to the MD simulation. In these four stages, the minimization procedure was applied to the following atoms of the system: (i) water molecules (2,500 steps); (ii) hydrogens atoms (2,500 steps); (iii) side chains of the amino acid residues and DNA (2,500 steps); and (iv) full system (10,000 steps). The minimized systems were then subject to a molecular dynamics equilibration procedure, which was divided into two stages: in the first stage (50 ps), the systems were gradually heated to 310.15 K using a Langevin thermostat at constant volume (NVT ensemble); in the second stage (50 ps), the density of the systems was further equilibrated at 310.15 K.

Finally, molecular dynamic production runs were performed for 300 ns. These were performed with an NPT ensemble at constant temperature (310.15 K, Langevin thermostat) and pressure (1 bar, Berendsen barostat), with periodic boundary conditions, with an integration time of 2.0 fs using the SHAKE algorithm to constrain all covalent bonds involving hydrogen atoms. A 10 Å cutoff for nonbonded interactions was used during the entire molecular simulation procedure. Coordinates were saved at each 10 ps.

Final trajectories were analyzed in terms of backbone RMSD, RMSF, and interactions formed.

### MM-GBSA

The molecular Mechanics/Generalized Born Surface Area method (35) was applied to estimate the histone-DNA-binding free energies in the presence and absence of doxorubicin. From each MD trajectory, a total of 500 conformations taken from the last 200 ns of simulation were considered for each MM-GBSA calculation.

According to the MM-GBSA method the binding free energy can be decomposed as the sum of different energy terms, defined as:


$$\Delta G_{bind}=G_{nucleosome}-(G_{histones}+G_{DNA})$$

$$\Delta G_{bind}=\Delta H-T\Delta S\approx \Delta E_{gas}+\Delta G_{sol}-T\Delta S$$

Because the structures of dimer and monomers or complex, protein, and ligand are extracted from the same trajectory, the internal energy change (∆*E*_int_) is canceled.


$$\Delta E_{gas}=\Delta E_{int}+\Delta E_{ELE}+\Delta E_{VDW}$$

$$\Delta G_{sol}=\Delta G_{GB}+\Delta G_{Surf}$$

The gas-phase interaction energy (∆*E*_gas_) between the components is written as the sum of electrostatic (∆*E*_ELE_) and van der Waals (∆*E*_VDW_) interaction energies. The solvation free energy (∆*G*_sol_) is divided into the polar and non-polar energy terms. The polar solvation energy (∆*G*_GB_) is calculated by using the generalized-born (GB) model. In this case, the GB model proposed by Onufriev, Bashford, and Case was considered (36). The non-polar contribution is calculated based on the solvent-accessible surface area (∆*G*_Surf_), calculated in the present work with the LCPO method (37). The calculated binding free energy (∆*G*_bind_) is, hence, written as the sum of the gas-phase interaction energy and solvation free energy.

### Infectivity assays

PBMCs were isolated from blood samples using Ficoll-Hypaque gradient centrifugation. After separation, PBMCs were pelleted by centrifugation. The cell culture medium consisted of RPMI 1640 supplemented with 20% heat-inactivated fetal bovine serum, 5% interleukin-2 (IL-2), and 50 µg gentamicin/mL. PBMCs (2 × 10^6^) isolated from whole blood were incubated with various concentrations of RS-1 (0, 15, 30, 75, and 100 µM) for 24 h at 37°C. Next, PBMCs were harvested (7 min, 400 × *g*) and resuspended in 500 µL culture medium. Ten microliters of HIV-1 subtype B virus (MOI = 0.1) was added to PBMC, and cells were incubated at 37°C for 3 h. Then, the medium was removed, and 10 mL of RPMI 1640 was added to wash the cells. The cells were harvested at low speed (400 × *g*), and the washed-cell pellet was resuspended in 2 mL of supplemented RPMI 1640. The cell suspension was added to wells of a 24-well tissue culture plate and incubated at 37°C. HIV-1 RNA from plasma samples was determined at 24, 48, and 72 h postinfection. Replication in PBMC was quantified by HIV-1 RNA determination in cellular supernatant using Amplicor HIV Cobas TaqMan, version 2 (Roche, Basel, Switzerland), with a lower limit of detection of 20 copies/mL of plasma.

vDNA quantifications were performed as previously described (38). Cells were harvested at different time post infection by centrifugation of 2 × 10^6^ to 10 × 10^6^ cell aliquots, and cell pellets were kept frozen at −80°C until further analysis. Total DNA (including integrated HIV-1 DNA and episomal HIV-1 DNA) was extracted using the QIAmp blood DNA minikit (Qiagen, Courtaboeuf, France) according to the manufacturer’s protocol. Elution was performed in 50 µL of elution buffer. The total HIV-1 DNA was amplified by quantitative real-time PCR using the Light Cycler instrument (Roche Diagnostics, Meylan, France). Amplification was performed in a 20-µL reaction mixture containing 1 × Light Cycler Fast Start DNA master hybridization probes (Roche Diagnostics), 3 mM MgCl_2_, 500 nM forward primer LTR152 (5′-GCCTCAATAAAGCTTGCCTTGA-3′, and 500 nM reverse primer LTR131 (5′-GGCGCCACTGCTAGAGATTTT-3′), located in an LTR region with highly conserved fluorogenic hybridization probe LTR1 (50 nM; 5′-6-carboxyfluorescein [FAM]-AAGTAGTGTGTGCCCGTCTGTT[AG]T[GT]TGACT-3′-6-carboxytetramethylrhodamine [TAMRA]). After an initial denaturation step (95°C for 10 min), total HIV-1 DNA was amplified for 45 cycles (95°C for 10 s, 60°C for 30 s), followed by 1 cycle at 40°C for 60 s. The copy number of total HIV-1 DNA was determined using the 8E5 cell line. The 8E5/LAV cell line, used for a standard curve, was derived from a CEM cellular clone containing a single, integrated, defective (in the *pol* open reading frame), constitutively expressed viral copy. 8E5 DNA (5 to 5 × 10^4^ copies) was amplified. Results were expressed as the copy number of total HIV-1 DNA per 10^6^ cells.

The 2-LTR DNA circles were amplified with primers HIV-F and HIV-R1, spanning the LTR-LTR junction, as described elsewhere (39). Briefly, amplification was performed in a 20-µL reaction mixture containing 1× Light Cycler Fast Start DNA master hybridization probes (Roche Diagnostics), 4 mM MgCl_2_, 300 nM forward and reverse primers spanning the LTR-LTR junction, and 200 nM each fluorogenic hybridization probe. Copy number of 2-LTR circles was determined in reference to a standard curve prepared by amplification of quantities ranging from 10 to 1 × 10^6^ copies of a plasmid comprising the HIV_LAI_ 2-LTR junction (39) by using Light Cycler quantification software, version 4.1 (Roche Diagnostics). Results are expressed as the copy number of 2-LTR circles per 1 × 10^6^ cells.

Integrated DNA was first amplified by Alu PCR performed in a 50-µL reaction mixture containing 200 ng total DNA; 1 × HF Phusion mix; 200 nM deoxynucleoside triphosphates (dNTP); 500 nM primer PBS (5′-TTTCAAGTCCCTGTTCGGGCGCCA-3′), located in the PBS sequence of the viral genome; and 500 nM primer Alu-164 (5′-TCCCAGCTACTGGGGAGGCTGAGG-3′), located in the Alu sequence of the cellular genome. After an initial denaturation step (98°C for 30 s), the heterogeneously sized population of integrated DNA was amplified for 35 cycles (98°C for 10 s, 60°C for 20 s, 72°C for 2 min 30 s). A second nested PCR was then performed in a 50-µL reaction mixture containing 5 µL of Alu PCR product, 1 × HF Phusion mix, 500 nM dNTP, 500 nM primer NI-1 (5′-CACACACAAGGCTACTTCCCT-3′), and 500 nM primer NI-2 (5′-GCCACTCCCCAGTCCCGCCC-3′); primer sequences match sequences localized in the viral genome. After an initial denaturation step (94°C for 12 min), the expected 351 bp fragment of integrated DNA was amplified for 42 cycles (94°C for 1 min, 60°C for 1 min, 72°C for 1 min). Results were analyzed on 1.2% agarose SYBR safe stain gel. A first-round PCR control was run in the absence of polymerase in order to quantify any unspecific amplification during the second round. The copy number of integrated HIV-1 DNA was determined in reference to a standard curve generated by concomitant two-stage PCR amplification of a serial dilution of the standard HeLa R7 Neo cell DNA mixed with uninfected-cell DNA to yield 50,000 cell equivalents. Cell equivalents were calculated according to the amplification of the β-globin gene (two copies per diploid cell) with commercially available materials (Control Kit DNA; Roche Diagnostics). 2-LTR circles and total and integrated HIV-1 DNA levels were determined as copy numbers per 10^6^ cells. Two-LTR circles and integrated cDNA were also expressed as a percentage of the total vDNA.

### BLaM virus fusion assay

Measurement of virus cell fusion efficiency was performed using chimeric viruses containing a Vpr protein fused with the β-lactamase (Vpr-BLaM) (40). MT4R5 cells were treated 30 min with doxorubicin or CA3 and infected with different doses (ng of p24) of a Vpr-BLaM containing NL-4.3 or NL-4.3Δenv-VSV-G viruses for 4 h at 37°C in the presence or absence of enfuvirtide (T20) at 5 µM. Cells were washed and incubated for 2 h with CO2-independent medium supplemented with 10% FCS and CCF2 (LiveBLAzer FRET-B/G loading kit with CCF2-AM, Thermo-Fisher Scientific). Cells were washed and fixed with 2% PFA, and the fluorescence intensity of cleaved and uncleaved CCF2 was measured by flow cytometry on a BD LSRII. Data were analyzed using the FlowJo 10 Software (BD Biosciences).

## Data Availability

All data are available upon request.

## SUPPLEMENTAL MATERIAL

Supplemental figures mbio.01083-23-s0001.pdf Figures S1-S10.

Legends mbio.01083-23-s0002.pdf Legends for supplemental figures.

ASM does not own the copyrights to Supplemental Material that may be linked to, or accessed through, an article. The authors have granted ASM a non-exclusive, world-wide license to publish the Supplemental Material files. Please contact the corresponding author directly for reuse.
