# Supplementary material for: Modulation of the functional interfaces between retroviral intasomes and the human nucleosome
Source: mBio. 2023 Jun 29;14(4):e01083-23. doi: 10.1128/mbio.01083-23 (PMC10470491; doi:10.1128/mbio.01083-23)
Supplement: Supplemental figures — Figures S1-S10. [file mbio.01083-23-s0001.pdf]

Sup data 1

A-

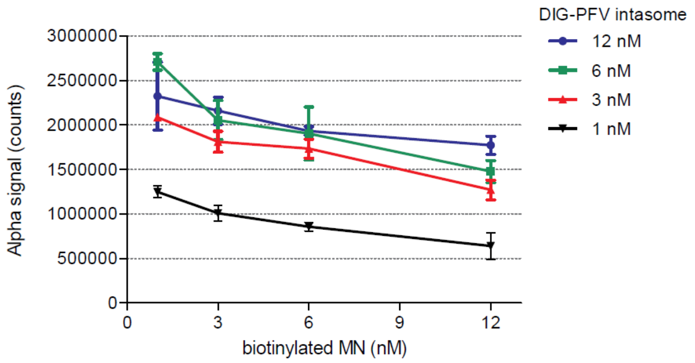
DIG-PFV intasome
12 nM
6 nM
3 nM
1 nM
Alpha signal (counts)
3000000
2500000
2000000
1500000
1000000
500000
0
0
3
6
9
12
biotinylated MN (nM)

B-

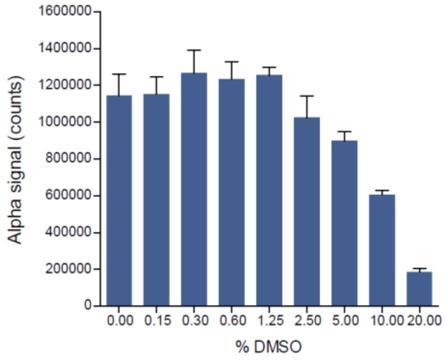
Alpha signal (counts)
1600000
1400000
1200000
1000000
800000
600000
400000
200000
0
0.00
0.15
0.30
0.60
1.25
2.50
5.00
10.00
20.00
% DMSO

Sup data 2

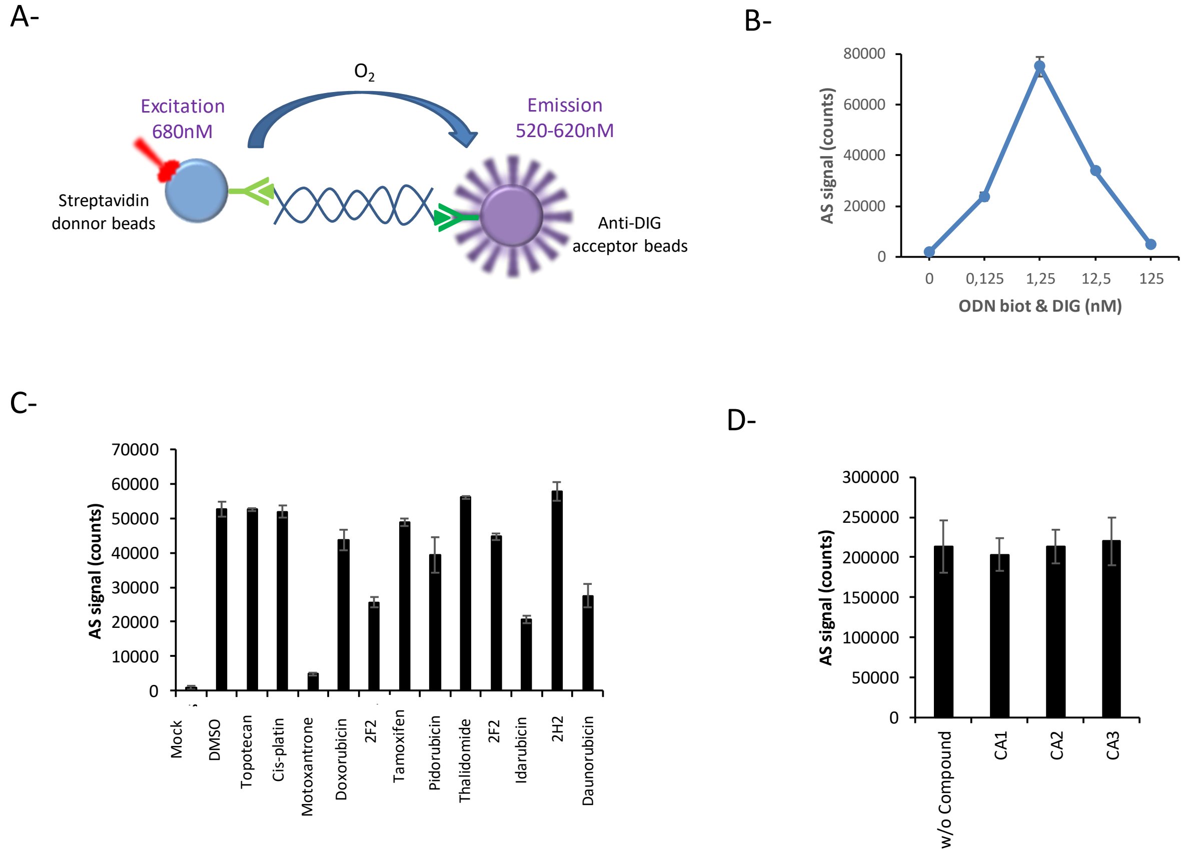
A-
O_2
Excitation
680nM
Emission
520-620nM
Streptavidin
donnor beads
Anti-DIG
acceptor beads
B-
80000
60000
40000
20000
0
AS signal (counts)
0
0,125
1,25
12,5
125
ODN biot & DIG (nM)
C-
70000
60000
50000
40000
30000
20000
10000
0
AS signal (counts)
Mock
DMSO
Topotecan
Cis-platin
Motoxantrone
Doxorubicin
2F2
Tamoxifen
Pidorubicin
Thalidomide
2F2
Idarubicin
2H2
Daunorubicin
D-
300000
250000
200000
150000
100000
50000
0
AS signal (counts)
w/o Compound
CA1
CA2
CA3

Sup data 3

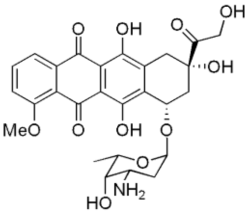

Doxorubicin

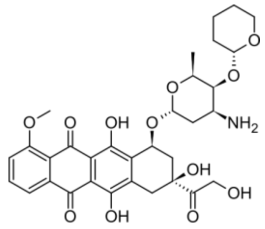

Pirarubicin

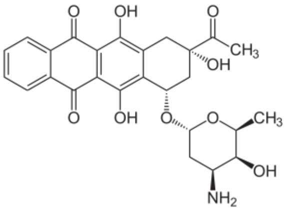

Idarubicin

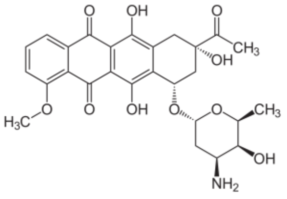

Daunorubicin

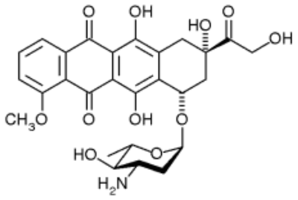

Isorubicin

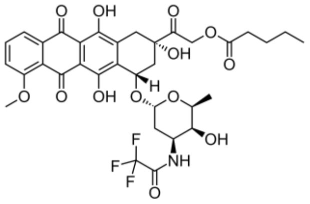

Valrubicin

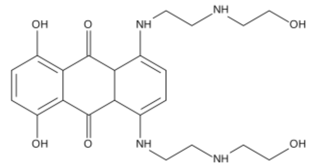

Mitoxantrone

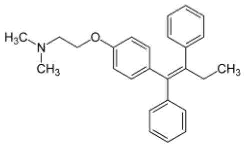

Tamoxifen

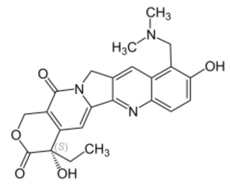

Topotecan

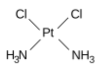

Cisplatin

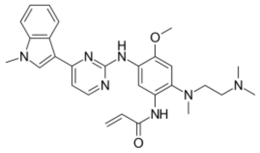

Osimertinib

Sup data 4

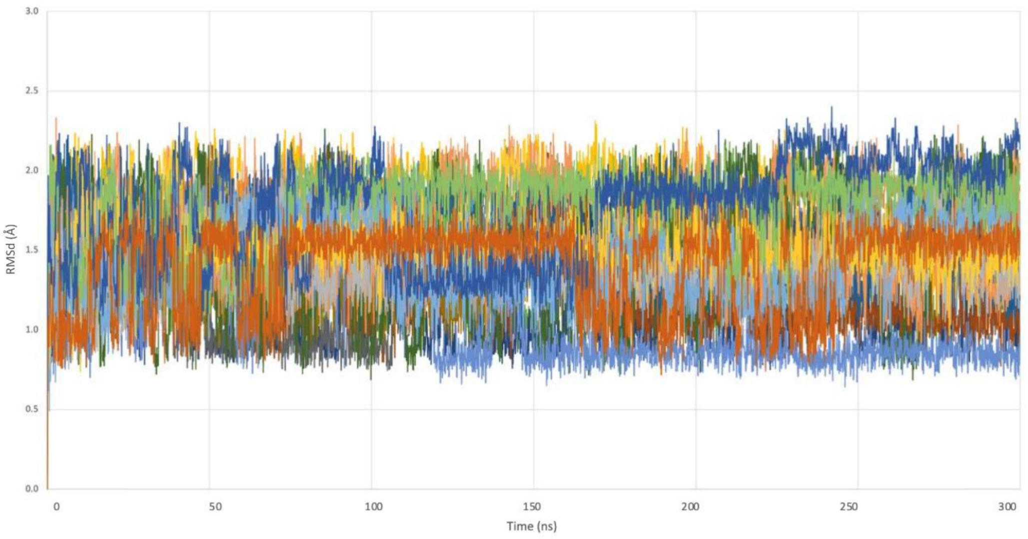

RMSd (Å)
Time (ns)

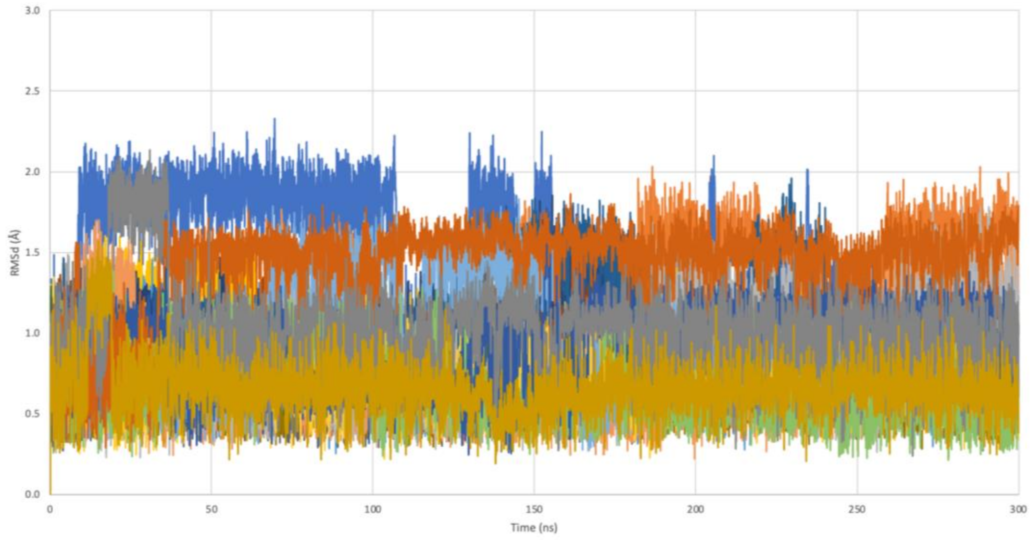

RMSd (Å)
Time (ns)

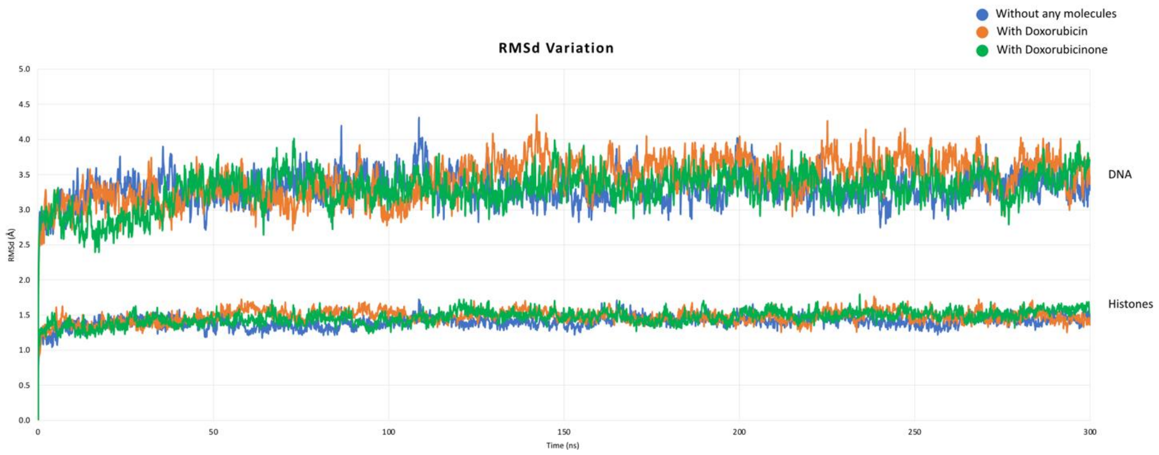

Without any molecules
With Doxorubicin
With Doxorubicinone
RMSd Variation
DNA
Histones
RMSd (Å)
Time (ns)

Sup data 5

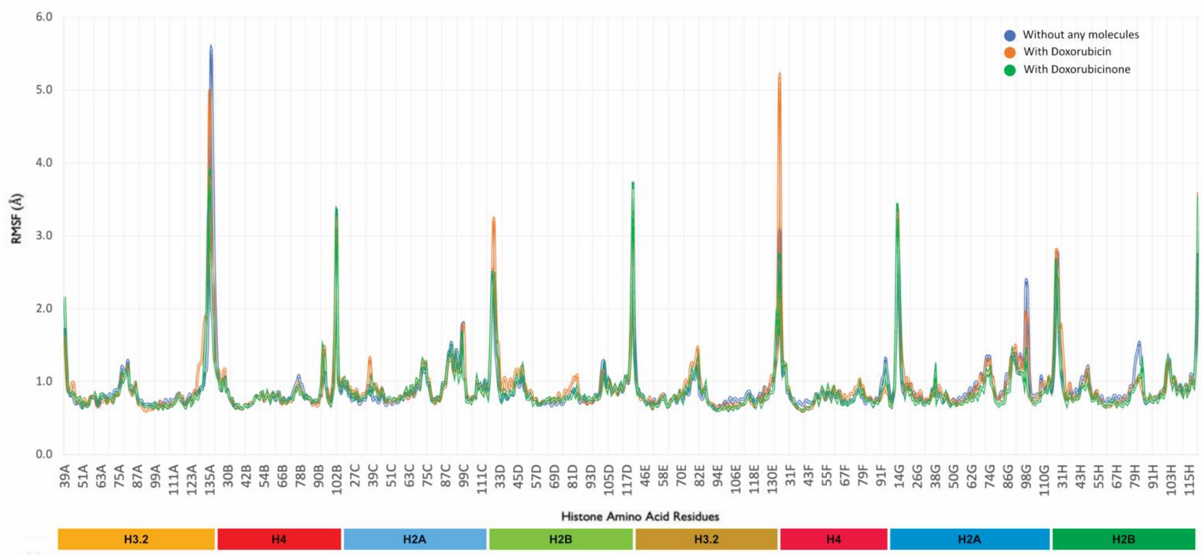

Without any molecules
With Doxorubicin
With Doxorubicinone
RMSF (Å)
6.0
5.0
4.0
3.0
2.0
1.0
0.0
39A 51A 63A 75A 87A 99A 111A 123A 135A 30B 42B 54B 66B 78B 90B 102B 27C 39C 51C 63C 75C 87C 99C 111C 33D 45D 57D 69D 81D 93D 105D 117D 46E 58E 70E 82E 94E 106E 118E 130E 31F 43F 55F 67F 79F 91F 14G 26G 38G 50G 62G 74G 86G 98G 110G 31H 43H 55H 67H 79H 91H 103H 115H
Histone Amino Acid Residues
H3.2
H4
H2A
H2B
H3.2
H4
H2A
H2B

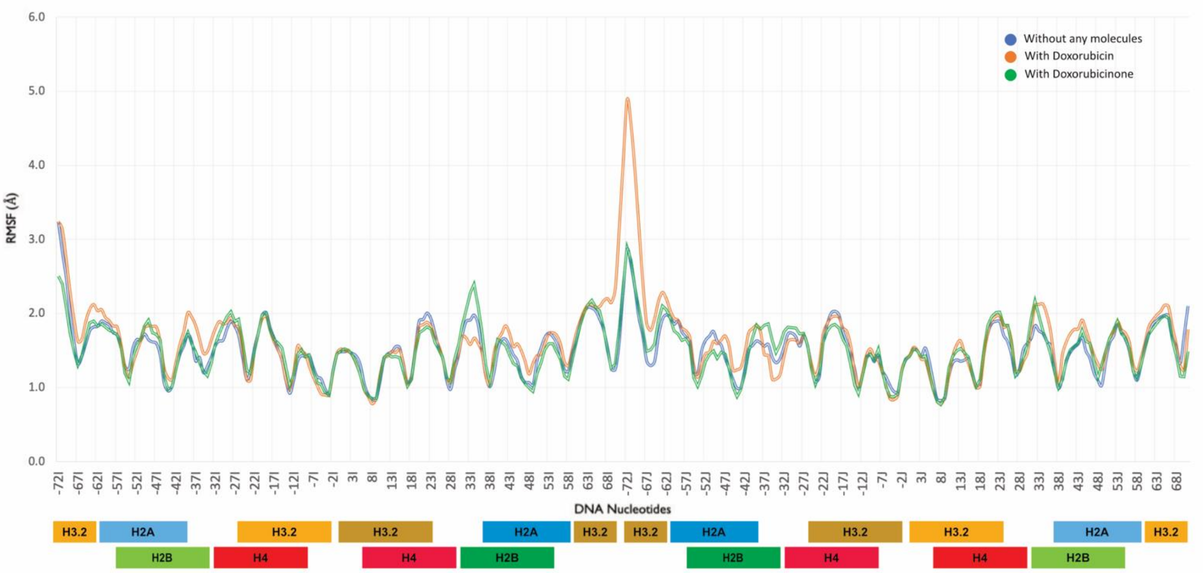

Without any molecules
With Doxorubicin
With Doxorubicinone
RMSF (Å)
6.0
5.0
4.0
3.0
2.0
1.0
0.0
-72I -67I -62I -57I -52I -47I -42I -37I -32I -27I -22I -17I -12I -7I -2I 3I 8I 13I 18I 23I 28I 33I 38I 43I 48I 53I 58I 63I 68I -72J -67J -62J -57J -52J -47J -42J -37J -32J -27J -22J -17J -12J -7J -2J 3J 8J 13J 18J 23J 28J 33J 38J 43J 48J 53J 58J 63J 68J
DNA Nucleotides
H3.2
H2A
H3.2
H3.2
H2A
H3.2
H3.2
H2A
H3.2
H3.2
H2A
H3.2
H2B
H4
H4
H2B
H2B
H4
H4
H2B

Sup data 6

A

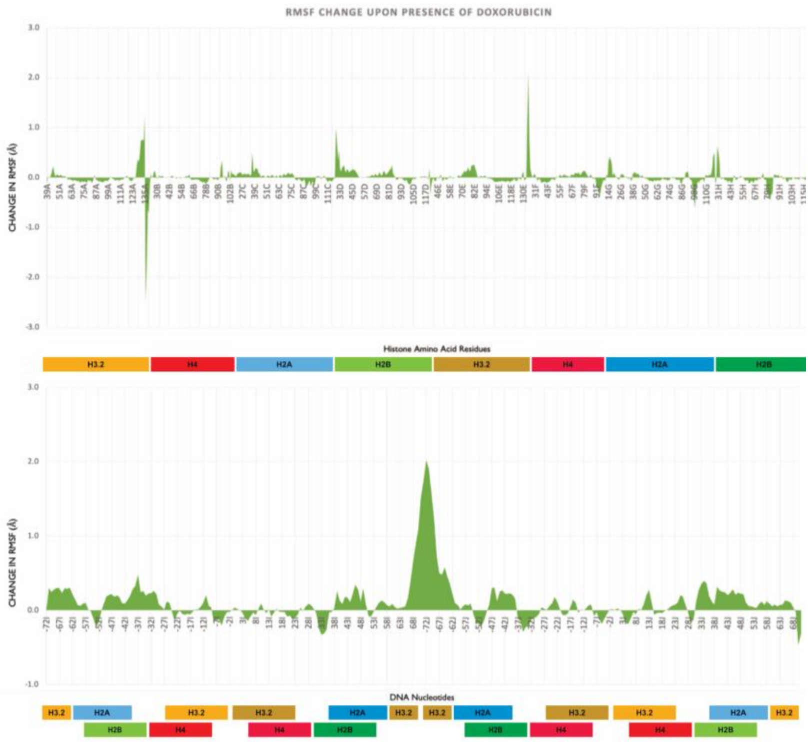
RMSF CHANGE UPON PRESENCE OF DOXORUBICIN
CHANGE IN RMSF (Å)
Histone Amino Acid Residues
H3.2
H4
H2A
H2B
H3.2
H4
H2A
H2B
CHANGE IN RMSF (Å)
DNA Nucleotides

B

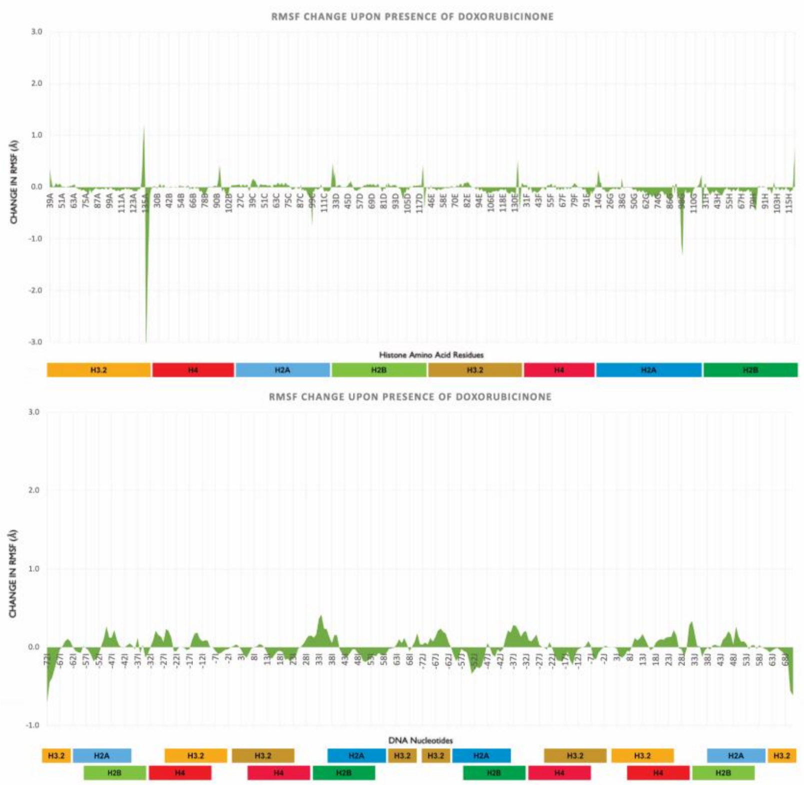
RMSF CHANGE UPON PRESENCE OF DOXORUBICINONE
CHANGE IN RMSF (Å)
Histone Amino Acid Residues
H3.2
H4
H2A
H2B
H3.2
H4
H2A
H2B
RMSF CHANGE UPON PRESENCE OF DOXORUBICINONE
CHANGE IN RMSF (Å)
DNA Nucleotides

C

H-bond donor	H-bond acceptor	Occupancy	Glycan Interaction
DOX 13	-51 J	85.8%	No
DOX 10	-8 I	59.4%	No
DOX 15	-47 J	52.5%	No
DOX 16	13 I	41.4%	No
DOX 17	52 I	41.2%	No
DOX 16	11 I	22.4%	Yes
DOX 7	-8 J	20.9%	No
-11 J	DOX 16	19.7%	Yes
DOX 1	72 J	19.3%	Yes
DOX 8	LYS43 H	17.6%	Yes
DOX 9	LYS43 H	16.9%	No
DOX 8	LYS43 H	15.0%	Yes
GLN24 G	DOX 9	13.2%	Yes
DOX 10	-7 I	13.0%	Yes
DOX 11	-9 I	11.7%	Yes
DOX 1	-71 I	11.1%	Yes
-72 J	DOX 18	10.6%	No

Sup data 7

A-

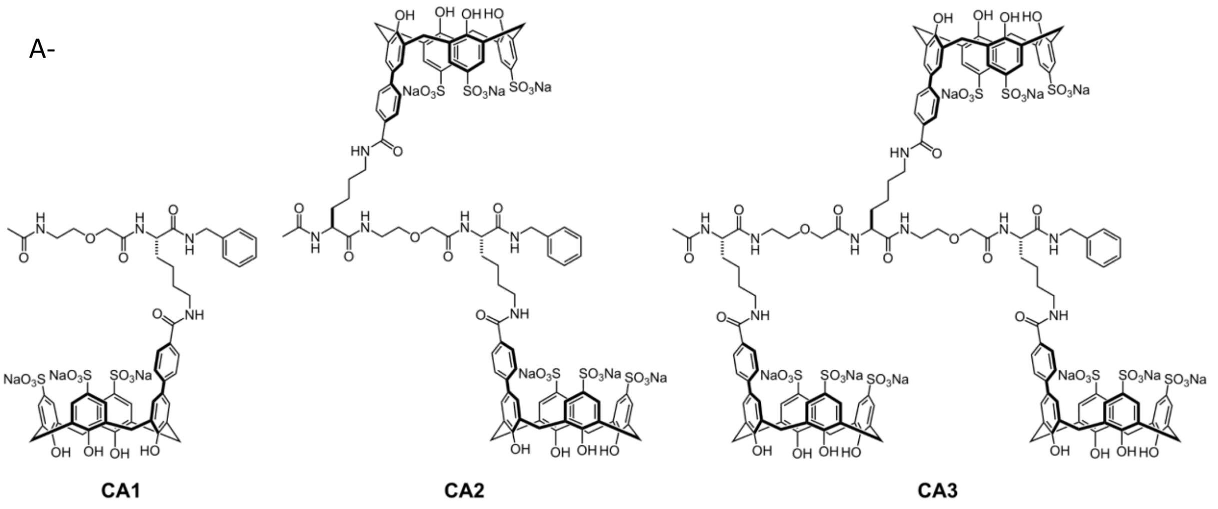

B-

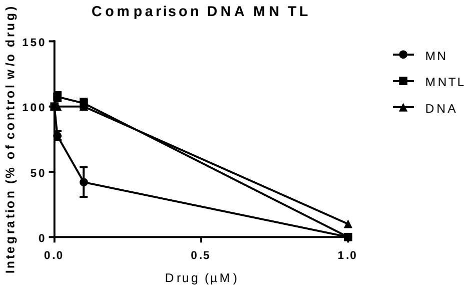

IC_{50} MN=0.0350-0.0920 ±0.0115 µM
IC_{50}MNTL=0.127-0.913 ±0.184 µM
IC_{50}Naked DNA=0.178-0.810 ±0.154 µM

Sup data 8

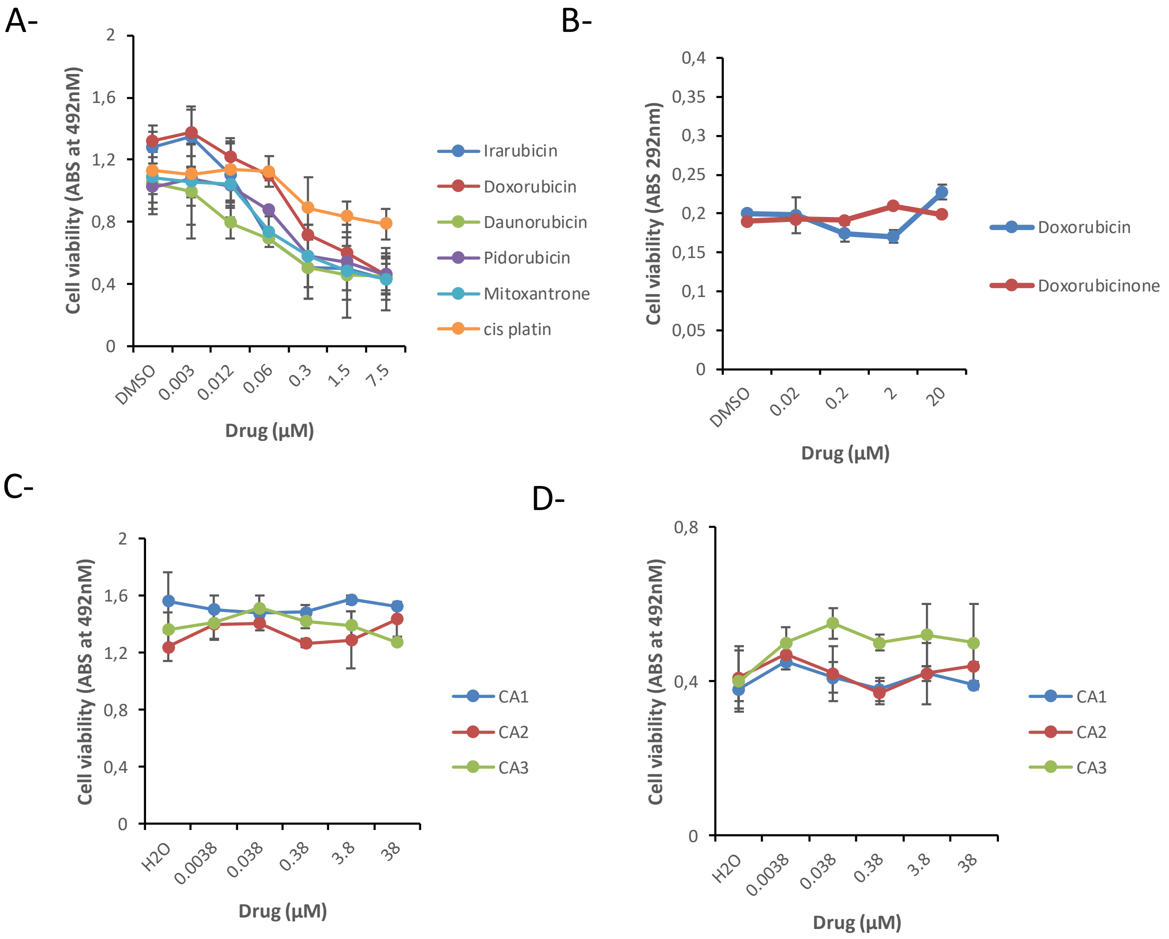
A-
Cell viability (ABS at 492nM)
2
1,6
1,2
0,8
0,4
0
DMSO
0.003
0.012
0.06
0.3
1.5
7.5
Drug (µM)
Irarubicin
Doxorubicin
Daunorubicin
Pidorubicin
Mitoxantrone
cis platin
B-
Cell viability (ABS 292nm)
0,4
0,35
0,3
0,25
0,2
0,15
0,1
0,05
0
DMSO
0.02
0.2
2
20
Drug (µM)
Doxorubicin
Doxorubicinone
C-
Cell viability (ABS at 492nM)
2
1,6
1,2
0,8
0,4
0
H2O
0.0038
0.038
0.38
3.8
38
Drug (µM)
CA1
CA2
CA3
D-
Cell viability (ABS at 492nM)
0,8
0,4
0
H2O
0.0038
0.038
0.38
3.8
38
Drug (µM)
CA1
CA2
CA3

Sup data 9

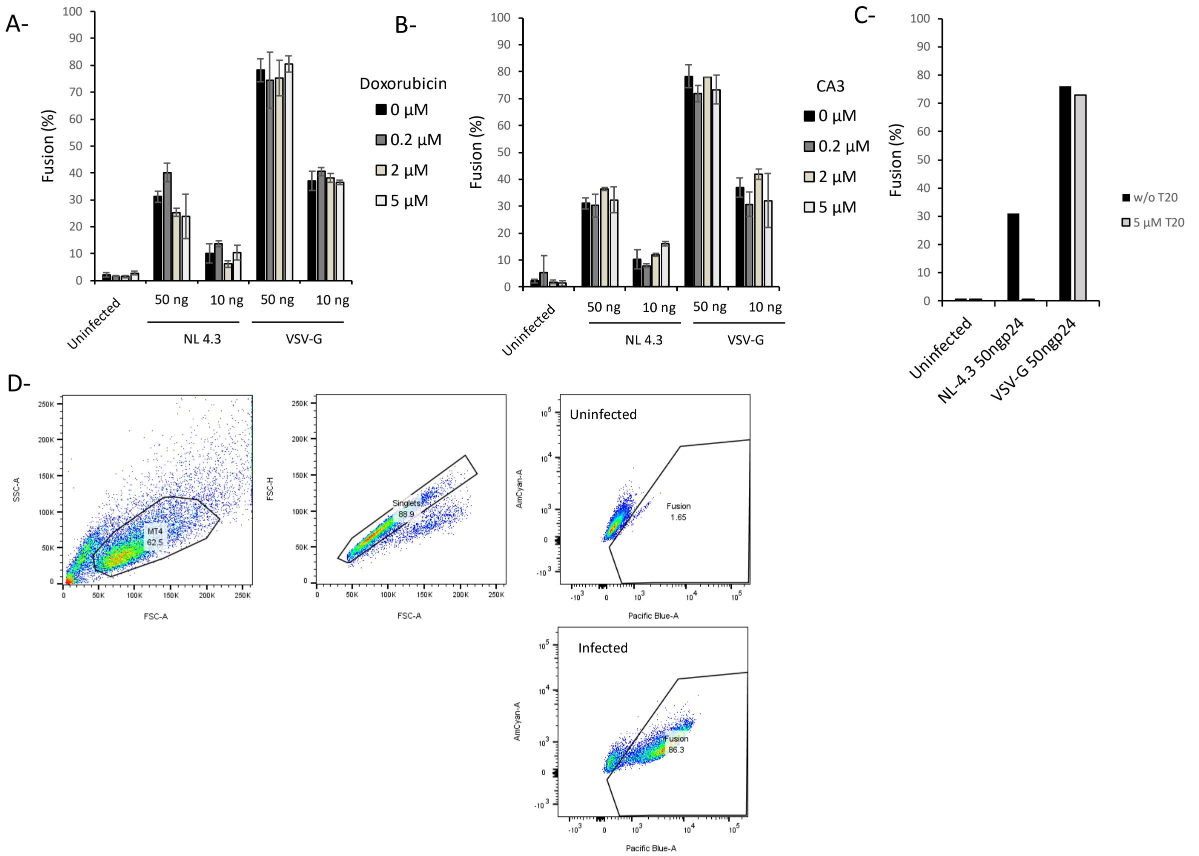

A-
Fusion (%)
Doxorubicin
0 µM
0.2 µM
2 µM
5 µM
Uninfected
50 ng
10 ng
NL 4.3
VSV-G
B-
CA3
C-
w/o T20
5 µM T20
NL-4.3 50ngp24
VSV-G 50ngp24
D-
SSC-A
FSC-A
MT4
62.5
FSC-H
Singlets
88.9
Uninfected
AmCyan-A
Pacific Blue-A
Fusion
1.65
Infected
Fusion
86.3

Sup data 10

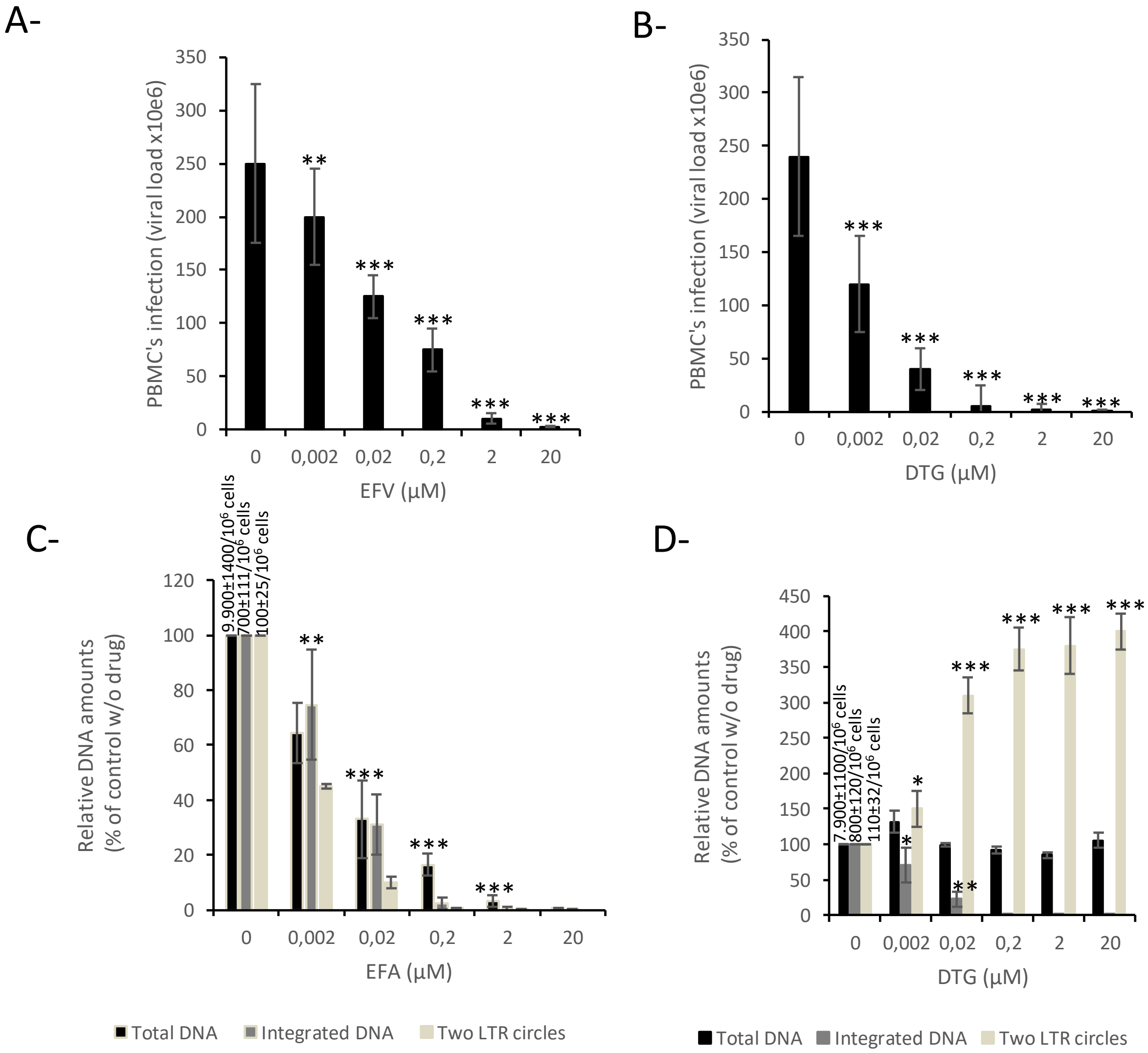
A-
PBMC's infection (viral load x10e6)
EFV (µM)
B-
PBMC's infection (viral load x10e6)
DTG (µM)
C-
9.900±1400/10^6 cells
700±111/10^6 cells
100±25/10^6 cells
Relative DNA amounts (% of control w/o drug)
EFA (µM)
Total DNA
Integrated DNA
Two LTR circles
D-
7.900±1100/10^6 cells
800±120/10^6 cells
110±32/10^6 cells
Relative DNA amounts (% of control w/o drug)
DTG (µM)
Total DNA
Integrated DNA
Two LTR circles
