# Supplementary material for: Modulation of the functional interfaces between retroviral intasomes and the human nucleosome
Source: mBio. 2023 Jun 29;14(4):e01083-23. doi: 10.1128/mbio.01083-23 (PMC10470491; doi:10.1128/mbio.01083-23)
Supplement: Legends — for supplemental figures. [file mbio.01083-23-s0002.pdf]

## SUPPLEMENTARY MATERIALS LEGENDS

**Sup data 1. AlphaLisa setup. A- Cross titration assay.** The Alphascreen interaction signal (AU) was monitored using increasing concentration of each partner and data are reported as mean of three independent experiments ±SD. **B-Effect of DMSO on PFV intasome binding to the nucleosome in AlphaLisa assay.** The Alphascreen interaction signal (AU) was monitored using increasing concentration of DMSO using 3nM of each partner. Data are reported as mean of three independent experiments ±SD.

**Sup data 2. Selectivity of the drugs and counter select assay.** The counter select assay was performed using short DNA fragment carrying a biotin and a DIG tag on each side (**A**). The Alphascreen interaction signal (AU) was monitored using increasing concentration the double tagged DNA (**B**). Each selected compound was then tested in optimized conditions at 10µM (**C**). Additional controls have been reported as non-selected drugs. CA1, CA2 and CA3 have also been tested in the counter select assay. Data are reported as mean of three independent experiments ±SD.

**Sup data 3. Chemical structures of the ONCOSET drugs used in this work.**

**Sup data 4. A- Representation of the RMSd for each of the doxorubicin molecules considered in the nucleosome simulations. B - Representation of the RMSd for each of the doxorubicinone molecules considered in the nucleosome simulations. C- RMSd variation of the backbone atoms of the nucleosome along the MD simulations for simulations without any molecules, with doxorubicin and with doxorubicinone.**

**Sup data 5. RMSF variation of the different residues and nucleotides of the nucleosome in the simulations done in the absence of any molecules, with doxorubicin and with doxorubicinone.**

**Sup data 6. Change in RMSF upon addition of doxorubicin (A) and doxorubicinone (B) for the histone amino acid residues and DNA nucleotides. (C) Principal hydrogen bonds between doxorubicin molecules and the nucleosome.**

**Sup data 7. A- Chemical structure of calixarenes molecules CA1, CA2 and CA3. B- Effect of CA3 on *in vitro* concerted integration catalyzed by HIV-1 intasomes on mononucleosome (MN), tailless nucleosome (MNTL) or naked 601 DNA.** CA3 has been added to a typical *in vitro* concerted integration performed with HIV-1 IN, LEDGF/p75 cofactor, radiolabeled viral U5 end fragment and MN, MNTL or naked DNA. The integration products were monitored on 6-12 % gradient polyacrylamide gel and quantified. Data are reported as mean of 3-4 independent experiments ±SD and $IC_{50}$ were reported in the figure.

**Sup data 8. Cell toxicity of the drugs were tested in HEK293T (A-B) and PBMC cells (C-D).** Cells were treated 24h and viability was measured by MTT assay as indicated in Material and Methods section. Absorbance was measured at 492 nm and data are reported as mean of 3 independent experiments ±SD.

**Sup data 9. Effect of Doxorubicin and CA3 on viral fusion.** MT4R5 cells were treated with doxorubicin (**A**) or CA3 (**B**) and infected with different doses of Vpr-BLaM containing NL-4.3 or NL-4.3Δenv-VSV-G viruses (ng of p24) and the β-lactamase cleavage of CCF2 was assessed by flow cytometry. Efficacy of the BLaM assay was confirmed using Enfuvirtide (T20) on 50ng of p24 of each viruse (a representative experiment is shown in **C**). Data are reported as mean of 3 independent experiments ±SD. Gating strategy of Vpr-BLaM assay was checked in **D.** MT4R5 cells are gated according to their structure, doublets are eliminated, and fusion gate is placed regarding the uninfected sample.

**Sup data 10. Effect of efavirenz and dolutegravir on HIV-1 replication.** PBMCs cells have been infected with HIV-1 virus in the presence of increasing concentrations of drugs. Replication was quantified by HIV-1 RNA determination in cellular supernatant (**A-B**). The viral DNA copy number per $10^6$cells has been reported in the figure. Viral DNA populations have been quantified by qPCR (**C**-**D**). Data are reported as mean of 3 independent experiments ±SD. *p<0.05, **p<0.005, ***p<0.001.
